# Glutamate-system defects behind psychiatric manifestations in a familial hemiplegic migraine type 2 disease-mutation mouse model

**DOI:** 10.1038/srep22047

**Published:** 2016-02-25

**Authors:** Pernille Bøttger, Simon Glerup, Bodil Gesslein, Nina B. Illarionova, Toke J. Isaksen, Anders Heuck, Bettina H. Clausen, Ernst-Martin Füchtbauer, Jan B. Gramsbergen, Eli Gunnarson, Anita Aperia, Martin Lauritzen, Kate L. Lambertsen, Poul Nissen, Karin Lykke-Hartmann

**Affiliations:** 1 Aarhus University, Department of Biomedicine, DK-8000 Aarhus, Denmark; 2 Centre for Membrane Pumps in Cells and Disease-PUMPKIN, Danish National Research Foundation, Aarhus University, Department of Molecular Biology and Genetics, DK-8000 Aarhus C, Denmark; 3 University of Southern Denmark, Institute of Molecular Medicine, Department of Neurobiology Research, DK-5000 Odense, Denmark; 4 The Lundbeck Foundation Research Centre MIND, Aarhus University, Department of Biomedicine, DK-8000 Aarhus C, Denmark; 5 University of Copenhagen, Department of Neuroscience and Pharmacology and Center for Healthy Aging, DK-2200 Copenhagen N, Denmark; 6 Karolinska Institutet, Department of Women’s and Children’s Health, SE-171 76 Stockholm, Sweden; 7 Aarhus University, Department of Molecular Biology and Genetics, DK-8000 Aarhus, Denmark; 8 Glostrup Hospital, Department of Clinical Neurophysiology, DK-2600 Glostrup, Denmark; 9 Danish Research Institute for Translational Neuroscience-DANDRITE, Nordic-EMBL Partnership of Molecular Medicine, Aarhus University, Department of Molecular Biology and Genetics and Department of Biomedicine, DK-8000 Aarhus C, Denmark; 10 Aarhus Institute of Advanced Studies, Aarhus University, Høegh-Guldbergs Gade 6B DK-8000 Aarhus C, Denmark

## Abstract

Migraine is a complex brain disorder, and understanding the complexity of this prevalent disease could improve quality of life for millions of people. Familial Hemiplegic Migraine type 2 (FHM2) is a subtype of migraine with aura and co-morbidities like epilepsy/seizures, cognitive impairments and psychiatric manifestations, such as obsessive-compulsive disorder (OCD). FHM2 disease-mutations locate to the *ATP1A2* gene encoding the astrocyte-located α_2_-isoform of the sodium-potassium pump (α_2_Na^+^/K^+^-ATPase). We show that knock-in mice heterozygous for the FHM2-associated G301R-mutation (α_2_^+/G301R^) phenocopy several FHM2-relevant disease traits e.g., by mimicking mood depression and OCD. *In vitro* studies showed impaired glutamate uptake in hippocampal mixed astrocyte-neuron cultures from α_2_^G301R/G301R^ E17 embryonic mice, and moreover, induction of cortical spreading depression (CSD) resulted in reduced recovery in α_2_^+/G301R^ male mice. Moreover, NMDA-type glutamate receptor antagonists or progestin-only treatment reverted specific α_2_^+/G301R^ behavioral phenotypes. Our findings demonstrate that studies of an *in vivo* relevant FHM2 disease knock-in mouse model provide a link between the female sex hormone cycle and the glutamate system and a link to co-morbid psychiatric manifestations of FHM2.

Familial Hemiplegic Migraine type 2 (FHM2) is a chronic and hereditary disorder with severe episodic attacks of migraine with aura (MA) fulfilling the classic migraine criteria^1^. FHM2 is a rare form of migraine with aura that involves motor aura (weakness) and frequently accompanied by co-morbid epilepsy/seizures^2^,^3^, and in a subset of FHM2-families cognitive impairments and/or different psychiatric manifestations such as mood depression, anxiety and obsessive compulsive disorder (OCD) have been reported^2^,^4^ as well as co-occurrence of obesity and other migraine forms^5^,^2^.

In the majority of FHM2-families, the disease is caused by haploinsufficiency due to loss-of-function mutations in the *ATP1A2-*gene, which encodes the α_2_-isoform of the Na^+^/K^+^-ATPase (a sodium pump)^2^,^6^. The sodium pump directs ion gradients (3Na^+^_out_/2K^+^_in_) which, in conjugation with other pump-independent functions, are implicated in various basic and specialized cellular functions. In the adult brain, the α_2_Na^+^/K^+^-ATPase is predominantly expressed in astrocytes^7^, and the astrocytic glutamate transporters of the EAAT family (excitatory amino acid transporters) uses the steep Na^+^-gradient maintained by the sodium pump as driving force for glutamate clearance from the synaptic cleft^8^,^9^,^10^. Moreover, the α_2_Na^+^/K^+^-ATPase do also co-localized with EAAT1 (GLAST) and EAAT2 (GLT-1)) in the astrocytic plasma membrane^11^,^12^.

Ovarian hormones significantly influence migraine^13^, and women have a higher prevalence of migraine after puberty, with a lifetime prevalence of 25% compared with 8% in men. A link between the female sex hormone cycle and the glutamate system was recently demonstrated since the selective estrogen receptor modulator (SERM) raloxifene was shown to upregulate EAAT2 and EAAT1 expression in rat primary astrocytes^14^.

One of the characteristic symptoms of FHM2 is the aura phenomenon appearing before the onset of the hemiplegic migraine. It is widely accepted that cortical spreading depression (CSD) is the molecular mechanism behind aura^15^. CSD gives rise to a propagating wave of neuronal and glial depolarization, which is accompanied by a massive distribution of ions between the extracellular space and intracellular department^16^. Two FHM1 knock-in mouse models^17^,^18^ and an *Atp1a2* mouse model harboring the W887R mutation^19^ revealed increased susceptibility to CSD compared to WT mice supporting CSD as a trigger to migraine. In this regard, it is noteworthy that while most of the *ATP1A2* mutations (and also the W887R mutation) are associated with pure FHM2, the *ATP1A2* G301R mutation represents a particular severe phenotype with an early onset.

In this study, we have generated an α_2_Na^+^/K^+^-ATPase knock-in (KI) mouse model (α_2_^+/G301R^) by introduction of the FHM2-associated G301R mutation described in two FHM2-families^4^,^20^. The α_2_^+/G301R^ mice displayed several behavioral phenotypes mimicking compulsive behavior and OCD, decreased sociability and stress-induced depression-like phenotypes. Interestingly, the α_2_^+/G301R^ mice displayed female-specific behaviors in several tests, and those behaviors—and the compulsive behaviors—were rescued by drug treatments targeting the female sex hormone cycle or the glutamate system. Altogether our results link the female sex hormone cycle and the glutamate system and a link to co-morbid psychiatric manifestations of FHM2.

## Materials and Methods

### Generation of the α_2_^+/G301R^ mouse line

#### Cloning of the targeting construct for generating α_2_^+/G301R^ mice

On the basis of 129/SvJ *Atp1a2-*gene fragments covering intron 6 to intron 13, the final targeting construct (total 16 691 bp) was generated using the pSC-B cloning vector as backbone (3.5 kb, Stratagene Corp, La Jolla, CA, USA). The targeting construct contained unmodified *Atp1a2-*gene stretches of 2.1 kb and 4.4 kb for cross-over (= recombination arms), a 2 × herpes simplex thymidine kinase (2 × TK) cassette (in *Sac*II site in multiple cloning site) conferring sensitivity to 1-(2′-deoxy-2′fluoro-1-ß-D-arabinofuranosyl)-5-iodouracil (FIAU), a floxed neomycin (NEO) gene cassette (in *Afl*II site in intron 7) conferring resistance to G418, and a modified *Atp1a2* genomic DNA stretch, which harbored a third LoxP site (abolishing a *Bst*EII site in intron 8) in addition to the G→A mutation in exon 8 (G→A in position 901 *post* start codon in mRNA, NCBI accession NM_178405, mouse strain C57BL/6) that introduced the G301R mutation. A detailed cloning strategy is listed in Supplementary Table 1. Note that the second of two LoxP sites in the targeting constructs was introduced for the possibility of generating conditional α_2_^+/KO^ mice (by crossing to Cre-expressing mice).

#### Gene targeting by homologous recombination

Murine 129S1/Sv-derived CJ7 embryonic cells^21^ were electroporated with *Pvu*I-linearized targeting construct, and G418-resistant (350 μg/mL) and FIAU-sensitive (0.5 μM) colonies were selected and expanded (DAGMAR facility, Aarhus University, Aarhus, Denmark (http://dagmar.au.dk)). Homolog recombination in ES cell clone IIH6 was confirmed by 5′end and 3′end PCR’s using primers located to the NEO cassette sequence and primers located to *Atp1a2* sequences flanking the *Atp1a2* sequences covered by the targeting construct, and a NEO PCR using the two NEO primers together (Supplementary Table 1). Moreover, homolog recombination in ES cell clone IIH6 was confirmed by Southern blotting with a probe binding to *Atp1a2* sequence flanking the *Atp1a2* sequence in the 5′end of the targeting construct (Supplementary Table 1) (DAGMAR facility). The NEO cassette was removed by partial Cre-enzyme treatment leaving a single LoxP site in intron 7 obtained by transfecting IIH6 ES cells with linearized Cre-enzyme encoding plasmid (DAGMAR facility). Successful partial Cre-enzyme treatment was confirmed for the IIH6Cre14 clone by PCR and digestions of the PCR product generated specific band patterns (Supplementary Table 1).

#### Generation and breeding of the transgenic α_2_^+/G301R^ knock-in (KI) mice

ES cells (IIH6cre14 clone) were individually injected into C57BL/6J blastocysts, which were then introduced into pseudo-pregnant female mice that gave birth to chimeric mice (DAGMAR facility). Two male chimeric mice were mated with C57BL/6J^BomTac^ (denoted C57BL/6J in this paper) female mice (Taconic Farms Inc, Bomholt, Denmark), and germ line transmission was obtained. Heterozygous α_2_^+/G301R^ mice were identified by PCR genotyping (Supplementary Table 1; protocol will be provided upon request) and subsequent *Bst*EII digestion, which generated a specific band pattern; note, that all mice were also retro-genotyped. In initial crossings between heterozygous α_2_^+/G301R^ male and female mice, homozygous α_2_^G301R/G301R^ pups were born, however they died neonatally in agreement with other mouse models targeting the *Atp1a2-*gene^19^,^22^,^23^,^24^. From four independent crossings, the α_2_^G301R/G301R^ mice, α_2_^+/G301R^ mice, and (α_2_^+/+^) littermates (WT) were born at the ratio 1:1:1 (9 α_2_^G301R/G301R^, 10 α_2_^+/G301R^, 10 WT) and generated fewer α_2_^+/G301R^ mice than expected according to Mendel’s law of segregation. All *in vivo* studies were performed using α_2_^+/G301R^ mice and WT obtained by crossing α_2_^+/G301R^ mice (generation N ≥ 8) with C57BL/6J mice (Taconic Farms Inc). Ten independent, and randomly chosen, breedings of α_2_^+/G301R^ mice with C57BL/6J mice that generated 80 offspring were assessed; the offspring distributed at the ratio 1:1 regarding sex (38 females, 42 males) and at the ratio 1:1 regarding genotype (43 α_2_^+/G301R^ (23 females, 20 males), 37 WT (15 females, 22 males), and showed that breeding generated offspring with a normal distribution of females and males and the expected distribution of genotypes (where genotypes were distributed at the ratio 1:1 regarding sex) according to Mendel’s law of segregation. The α_2_^+/G301R^ mice appeared grossly normal and were indistinguishable from WT mice by eye. Mice were housed in a room under a 12:12 light/dark cycle (lights on between 6:30A.M. and 6:30P.M.) in cages with littermates of same sex. Food and water were provided *ad libitum*. Experimental protocols involving mice, performed at Aarhus University and Copenhagen University, were done according to the Danish national and institutional regulations and approved by the Animal Experiments Inspectorate under the Danish Ministry of Justice (permit numbers 2012-15-2934-00621, 2013−15−2934−00815 and 2012-15-2934-00063). Moreover, experimental protocols involving mice, performed at Karolinska University, were done according to the European Communities Council Directive of 24 Nov. 1986 (86/609/EEC) and approved by the Northern Stockholm Laboratory Animal Review Board (permit number N132/12).

All behavioral experiments were done blind to genotype with age-matched littermates.

#### Body weight development

Each mouse was weighed every week from day 0 to day 190, and in intervals hereafter up to 360 days. The body weight (g) was determined with two decimals (FX-3000i, A&D Company Ltd, Tokyo, Japan). The data, given as body weights (g) at the given days, was shown as means and analyzed using two-way ANOVA, see Supplementary Table 1.

#### SDS-page Western blotting

Crossing of two heterozygous α_2_^+/G301R^ mice generated newborn pups of all genotypes (homozygotes (−/−), heterozygotes (+/−) and WT (+/+)) which were decapitated, and to each brain, 0.3 mL ice-cold lysis-buffer (1% sodium dodecyl sulfate (SDS), 1 mM Na_3_VO_4_, protease inhibitor cocktail (Complete^TM^ (mini, EDTA-free), 04 693 159 001, Roche Diagnostics GmbH, Mannheim, Germany), 10 mM Tris (pH 7.4)) was added, and next grinded 10 × using a 60 μm pestle in a glass rod (885301-0002 tissue grinder, Kimble Chase, Gerresheimer Glass Inc, Vineland, NJ, USA). The lysates were centrifuged 2 min, 1,000 × g at 4 °C, and the supernatants were aliquoted and samples were stored at −80 °C. Two adult male and female mice of each genotype were decapitated. The brains from two mice were dissected into hippocampus (HC), cortex (CTX), brain stem (BS) and cerebellum (CRBL) and the brain areas were stored on ice. Immediately hereafter, the brain areas from two mice were pooled and 0.4 mL (HC) or 0.8 mL (other) ice-cold lysis-buffer was added, and hereafter handling was as described above. Male and female α_2_^+/G301R^ mice were mated, and vaginal plug was noted. Pregnant female mice were sacrificed 17 days after conception, and the embryos (E17.5) were dissected, measured, and the embryonic stage was confirmed by the length (in cm) and their visual appearance. The embryos were decapitated and the whole brains were collected and to each brain, 0.3 mL ice-cold lysis-buffer was added, and hereafter handling was as described above. The protein content in all samples was determined using a method adapted from Bradford^25^ (protein assay kit 1, 500-001, Bio-Rad Laboratories Inc, Berkeley, CA, USA). In total, 25 or 30 μg of protein lysates in reducing sample-buffer (58 mM Tris (pH 6.8), 5% (v/v) glycerol, 1.7% (w/v) SDS, 0.002% (w/v) bromphenol blue, 100 mM dithiothreitol (DTT)) (no boiling) and a molecular weight standard marker (spectra^TM^ multicolor broad range protein ladder (SM1841, Fermentas, Thermo Fisher Scientific Inc, Waltham, MA, USA)) were separated by SDS-page using 10% uniform polyacrylamide gels and blotted onto 0.45 μm pore-sized polyvinylidene fluoride (PVDF) membranes (IPVH00010 Immobilon^R^-P, Millipore Corp, Bedford, MA, USA). The blots were blocked for 1 hour at room temperature (RT) in blocking solution (5% swine serum in phosphate-buffered saline (PBS (pH 7.4)) with 0.05% detergent (Tween-20) (PBS-T)). The blots were incubated with primary antibody (polyclonal rabbit anti-human α_2_ aa 432–455, 07674 Upstate, Millipore Corp, polyclonal rabbit anti-rat α_3_, aa 320–514, 06172 Upstate, Millipore Corp, and monoclonal mouse anti-chicken α_1_, polypeptide 1020 aa, developed by Douglas M. Fambrough, obtained from the Developmental Studies Hybridoma Bank developed under the auspices of the NICHD and maintained by The University of Iowa, Department of Biology, Iowa City, lA 52242) over-night at 4 °C, diluted 1:5000 (α_2_ antibody) and 1:1000 (α_1_ and α_3_ antibodies) in blocking solution, followed by 3 × 10 min wash-steps in PBS-T at RT. The blots were then incubated with secondary antibody (horseradish peroxidase (HRP)-conjugated polyclonal swine anti-rabbit IgG, P0217, Dako Inc, Glostrup, Denmark) for 1 hour at RT diluted 1:1 000 in 5% (w/v) nonfat dry milk in PBS-T, followed by 3 × 10 min wash-steps in PBS-T at RT. Note that blocking, incubations, and in-between washes were done on a rocking table. The blots were developed using a chemiluminescent substrate according to the manufacturer’s instructions (ECL^TM^ western blotting analysis system, RPN2109, Amersham^TM^, GE Healthcare, Little Chalfont, Buckinghamshire, UK) and visualization occurred by use of a scientific imaging system (LAS-3000 Luminescent Image Analyzer, Fuji film Corp, Kanagawa, Japan). After development, the blots were stripped at RT by incubating 15 min in H_2_O, 2 × 15 min in 0.1 M NaOH, 15 min in H_2_O, followed by 30 min in PBS-T. The blots were blocked for 1 hour at RT in blocking solution (5% swine serum in PBS-T). The blots were incubated with primary antibody (polyclonal rabbit anti-human glyceraldehyde 3-phosphate dehydrogenase (GAPDH) (aa 1–335, sc-25778, Santa Cruz Biotechnology Inc, Santa Cruz, CA, USA) over-night at 4 °C diluted 1:200 in blocking solution, followed by 3 × 10 min wash-steps in PBS-T at RT. The blots were then incubated with secondary antibody and developed as described above.

The SDS-page Western blotting experiments were performed at least twice using independent samples, and representative blots were subjected to densitometric analysis where the intensity of the bands was quantified as arbitrary units, AU or Quantum Level, QL (note that background stain in each lane was also determined in parallel and subsequently subtracted). Densitometry was performed using the Multi Gauge v3.2 software (Fuji film Corp), see Supplementary Table 1. The level of α_2_-protein relative to GAPDH in lysates made from WT E17.5 embryos and newborn pups was set to 100%. In the experiments where more than one WT lysate was tested, the specific level of α_2_-protein relative to GAPDH was normalized to the average level of α_2_-protein in WT lysates. The relative α_2_-protein level in lysates from the different genotypes in % is shown as means ± s.e.m (Supplementary Table 2). For adult Western blot quantification, ImageJ was used to obtain a numerical value corresponding to the intensity for each band of interest. The three α-isoform protein levels were measured relative to GAPDH for both genotypes and WT ratios were set to 100%. The relative α-isoform protein levels obtained for heterozygotes (+/−) are shown as means ± s.e.m. The specificity of the α_2_ antibody was confirmed on a Western blot of rat brain microsomal preparation and allowed for recognition of a protein of 105 kDa (company product sheet), and specificity of the GAPDH antibody was confirmed on a Western blot of HeLa and JAR cell lysates and mouse placenta tissue and allowed for recognition of a protein of 37 kDa (company product sheet).

### Electrophysiology

#### Surgery

**Figure 1:**
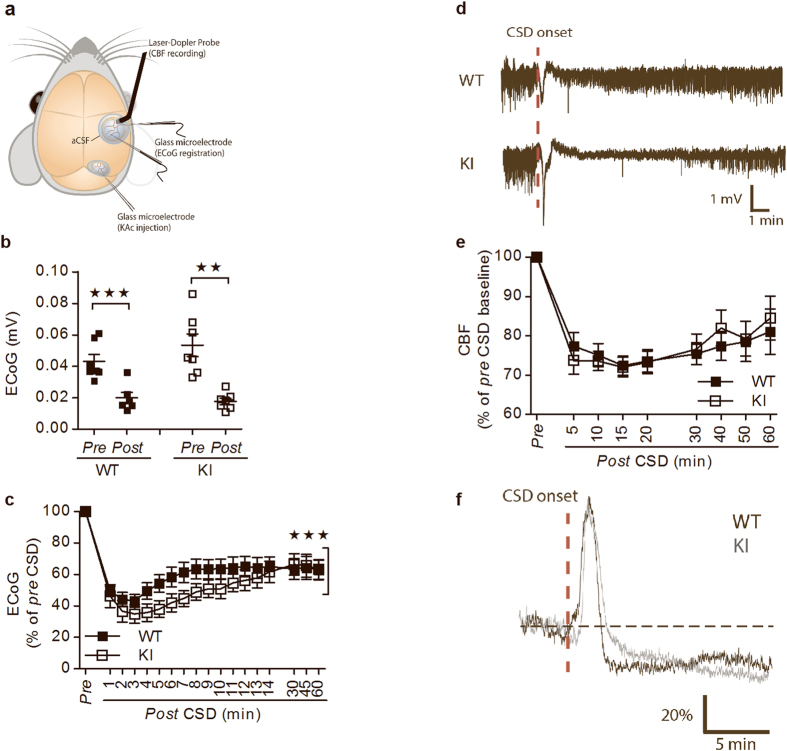
Cortical spreading depression affected the recovery of ECoG signal but not the cerebral blood flow. (**a**) Drawing illustrating the placement of laser-Doppler probe (cerebral blood flow), the glass microelectrodes for measuring local field potential (LFP)/ECoG recordings, and the KAc microinjection site (for cortical spreading depression (CSD)-induction). (**b**) Both male α_2_^+/G301R^ (n = 7) and WT (n = 6) mice showed an overall reduction in electrocorticography (ECoG) signal (mV) following CSD (~1.5 min *post* CSD). (**c**) ECoG signal (as % of *pre* CSD) showed that male α_2_^+/G301R^ mice (n = 6–7) recover more slowly after cortical spreading depression (*post* CSD) induction compared to WT mice (n = 6–7). (**d**) Representative examples of ECoG signal with DC (traces are shown for the first 15 min, Fig. 1d). (**e**) Male α_2_^+/G301R^ (n = 7) and WT mice (n = 7–8) show no difference in the cerebral blood flow (CBF as % of *pre* CSD baseline) after CSD-induction (*post* CSD). Data shown as means ± s.e.m. ★★★*P* < 0.001. (**f**) Representative examples of CBF signal in % of pre-CSD baseline are shown for the first 15 min. The CSD is followed by an acute hyperemia followed by an extended oligemia (Fig. 1 f). Note that the data was analyzed with 5/10 min interval, thus, the initial hyperemia is not seen in Fig. 1 f.

Mice were anesthetized by intraperitoneal (i.p.) injections of a mixture of ketamine (60 mg/kg) and xylazine (10 mg/kg) (Sigma-Aldrich Co, St Louis, MO, USA), and they were given supplemental doses of ketamine every 20 min. Body temperature was maintained at 37 °C using a temperature controller and a heating blanket (model TC-1000 Temperature Controller, CWE Inc, Ardmore, PA, USA). The trachea was cannulated for mechanical ventilation with a small-animal ventilator (SAAR-830, CWE Inc). Catheters (100828 DiLab, VeruTech AB, Lund, Sweden) were placed into the left femoral artery and vein and perfused with physiological saline. The end-expiratory CO_2_ (microCapstar End-tidal CO_2_ Monitor, CWE) and blood pressure (Pressure Monitor BP-1, World Precision Instruments, Sarasota, FL) were monitored continuously in combination with blood gases in arterial blood samples (pO_2_: 115–130 mmHg; pCO_2_: 35–40 mmHg; pH: 7.35–7.45; ABL 700 Series; Radiometer Medical, Brønshøj, Denmark) to ensure that animals were kept under physiological conditions. Two craniotomies were performed with a dental drill; one (2-mm-diameter) placed over the barrel cortex was used for recording, and the other (1-mm-diameter) placed over the rostral parietal lobe was used for elicitation of cortical spreading depression (CSD). The dura was carefully removed and visible signs of brain damage or bleeding excluded mice from the experiment. During and after removal of the dura, the craniotomy sites were continuously superfused with artificial cerebrospinal fluid (aCSF) (aCSF composition in mM: 2.0 glucose, 126.0 NaCl, 2.8 KCl, 22.0 NaHCO_3_, 1.45 CaCl_2_, 1.0 Na_2_HPO_4_, and 0.876 MgCl_2_). The craniotomies were covered with a drop of 0.75% agarose in saline. After the preparation and application of electrodes and probes, anaesthesia was changed to α-chloralose (50 mg/kg/hour, intravenous (i.v.), Sigma-Aldrich Co) that is the best regime to study cerebral blood flow^26^,^27^. For schematic drawing of setup, see Fig. 1a. Experiments were performed after a postoperative recovery period of at least 30 min in order to obtain a stable level of anaesthesia, a stable laser-Doppler baseline, and an arterial blood pressure of 80–110 mmHg. In the end, mice were killed by an overdose of anaesthesia and decapitated afterwards.

#### Induction of cortical spreading depression

CSD was elicited in the right somatosensory cortex by pressure injection of potassium acetate (KAc, 1 M), and propagated to the barrel cortex. KAc has the advantage over KCl that the tissue is similarly depolarized by K^+^ while Ac serves as an anion substitution decreasing the Cl^−^ concentration and hence the Cl^−^ gradient across the plasma membrane. To allow for induction of single CSD, a micropipette was filled with KAc, connected to a picopump (World Precision Instruments) and inserted into the craniotomy at a depth of approximately 200 μm. KAc was injected during 1–2 s with a 4–6 psi pressure. If no CSD was elicited within 3 min, the injection was repeated until a CSD was elicited. If no CSD was elicited after 3 trials, the injection electrode was retracted to verify that it had not been clogged, before injections continued. Spontaneous CSD events were observed in few mice (1:8 WT mice and 1:9 α_2_^+/G301R^ mice) after the first CSD event was induced. Notably, these rare spontaneous CSD events could be the result of e.g., remaining KAc in the tissue or KAc from a leaking electrode. The data, given as CSD propagation speed (for a single CSD event) (μm/s), was shown as mean ± s.e.m. and analyzed using Student’s two-tailed, unpaired *t*-test, see Supplementary Table 3.

#### Electrophysiological recording

The electric signals of the CSD were monitored by two glass microelectrode recording both electrocorticographic (ECoG) signal and the direct current (DC). The DC was recorded to follow the voltage shift during the CSD. ECoG signal constitutes the total electrical signal (without the DC offset) from the cortex surrounding the electrode and is composed of spikes and spontaneous local field potentials, where the largest component is low (<100 Hz) frequency signal. The glass microelectrodes used were single-barrelled, filled with 2 M saline. Two electrodes were placed at a depth of approximately 150 μm in the right somatosensory cortex and they were separated to allow for measurement of the CSD propagation speed. The DC signals were amplified using a differential amplifier (gain × 10, bandwidth 0.1–5 000 Hz, DP-311, Warner Instruments, Hamden, CT, USA), A/D converted (20 kHz) and low pass filtered (5 kHz). The ECoG signal was obtained by amplification of the DC signal using CyberAmp 380 (gain × 100, bandwidth 0.1–5 000 Hz; Axon Instruments Inc, Foster City, CA, USA); these signals were digitized using the 1401 plus hardware connected to a computer running the Spike 2.7 software (Cambridge Electronic Design). Stimulation of the mouse whisker pad was done to assess the evoked potentials. Mouse sensory barrel cortex was stimulated contralateral to the craniotomy, in the ramus infraorbitalis of the trigeminal nerve by a set of custom-made bipolar electrodes inserted percutaneously. The cathode was positioned corresponding to the hiatus infraorbitalis (IO) and the anode was inserted into the masticatory muscles. The thalamocortical IO stimulation was done with an intensity of 1.5 mA (ISO-flex, A.M.P.I., Israel) lasting 1 ms, in trains of 15 s at 0.5 Hz. The stimulation was controlled by a sequencer file running within the Spike2 software (version 7.02, Cambridge Electronic Design) and recorded with the same glass microelectrode as for ECoG signal. The amplitude of the local field potentials were summed over the duration of the stimulation (summa LFP) and presented as % relative to pre-CSD induction as means ± s.e.m. and analyzed using a linear mixed model, see Supplementary Table 3. Evoked potentials were recorded before the CSD and 15 min after the CSD (stimulations were not performed at 0–15 min in order to not perturb the ECoG signal with repeated stimulations which potentially could influence the rate of recovery of the ECoG signal).

The ECoG signal was root mean squared and averaged over periods of 1 min and presented as % of the total electrical signal relative to pre-CSD induction and as mV, were shown as means ± s.e.m. and analyzed using a linear mixed model, see Supplementary Table 3.

#### Cerebral blood flow measurements

CBF was recorded continuously using a laser-Doppler flowmetry (LDF) probe at a fixed position 0.3 mm above the pial surface in a region devoid of large vessels (wavelength 780 nm; 250 μm fiber separation allowing CBF measurement to a depth of 1 mm; Perimed AB, Järfälla, Sweden)^28^. The probe was placed close to one of the microelectrodes recording electrophysiological variables. The LDF signal was smoothed with a time constant of 0.2 s (PeriFlux 4001 Master, Perimed AB), sampled at 10 Hz, analog-to-digital (A/D) converted, and digitally recorded and smoothed (time constant 1 s) using the Spike2 software (Cambridge Electronic Design). The LDF method does not measure CBF in absolute terms, but is valid in determining relative changes in CBF during moderate flow increases^29^.

The CBF baseline was evaluated as % of pre-CSD baseline with a 5 min average value was calculated before CSD and a 1 min average value was calculated 5, 10, 15, 20, 30, 40, 50 and 60 min after CSD. Data is shown as means ± s.e.m. and analyzed using a linear mixed model, see Supplementary Table 3.

### Behavioral Analysis

#### Open field

Each individual mouse was placed in a bright grey, squared plastic box (45 × 45 × 45 cm), and the movement of the mouse was recorded using the SMART video tracking software system (version 2.5.20, Panlab Harvard Apparatus, Cornellà (Barcelona), Spain) connected to a video camera (SSC-DC378P, Sony Corp, Tokyo, Japan) placed directly above the box for 10 min^30^. The system automatically recorded the total distance travelled (m), mean velocity (cm/s) and time (s) spent in each zone (17.6 × 16.1 cm imaginary square). The number of groomings (n), urinations (n) and droppings (n) were counted manually. Each mouse was tested once, and the open field setup was cleaned with 70% ethanol and wiped with paper towels between each trial. The data, given as distance travelled (m), mean velocity (cm/s), faecal boli (n), urinations (n) and center/perimeter ratio, were shown as means ± s.e.m. and analyzed using Student’s two-tailed, unpaired *t*-test with Welch’s *post hoc* correction where indicated in Supplementary Table 4. Two-sided Fisher’s exact test was used where Student’s two-tailed, unpaired *t*-test was not applicable, see Supplementary Table 4.

#### Accelerating rotarod test

The rotating rod apparatus (Rota-rod/RS, PanLab Harvard Apparatus) was used to measure the ability of mice to improve motor skill performance/coordination with training. Mice were placed on the rod (3-cm-diameter) for three trials per day for four consecutive days. The first day consisted of 3 × training trials where no data were obtained. The next three days, the first trial of the day was a training trial where no data were obtained, whereas the following two trials were recorded using the SeDaCom software system (version 1.4.02, PanLab Harvard Apparatus). When a mouse fell off the rotarod, it was reintroduced to the rotarod once more, and if the latency to fall this time was more than 20 s, then this latency (s) was added to the latency to fall (s) obtained first. Each trial lasted a maximum of 5 min, and during this time, the rotating rod underwent a linear acceleration from 4 to 40 rpm. Thus, mice were scored for their latency to fall (s) for two trials a day, and the average of these latencies was the latency to fall (s) for a given mouse on that given day. Mice rested a minimum of 20 min between trials to avoid fatigue and exhaustion. The rotarod was cleaned with 70% ethanol and wiped with paper towels between each trial. The data, given as latencies to fall (s) at the indicated days, were shown as means ± s.e.m. and analyzed using two-way ANOVA, see Supplementary Table 4.

#### Grip strength

The total grip strength was measured using a grip strength meter (Bio-GT3, Bioseb, Panlab Harvard Apparatus). The mouse was allowed to grasp the metal grid with both front paws and then pulled backwards in the horizontal plane. The force at the moment when the mouse lost its grip was recorded as the peak tension given in grams (g). Each mouse was tested in 5 sequential trials, and the highest grip strength was recorded as the grip strength parameter^30^. In parallel, the body weight (g) of each mouse was determined. The data, given as grip strength (g) relative to body weight (g), were shown as means ± s.e.m. and analyzed using Student’s two-tailed, unpaired *t*-test with Welch’s *post hoc* correction, see Supplementary Table 4.

#### Estradiol levels in serum

Blood (approximately 200–300 μL) from individual mice was collected from the neck after decapitation and allowed to clot 30 min at RT. The samples were centrifuged at ~2 000 × g at 4 °C, 15 min. The serum was transferred to new tubes and the centrifugation step was repeated. Serum was aliquoted and stored at −80 °C. The estradiol assay protocol (Mouse/Rat Estradiol ELISA ES180S-100, Calbiotech Inc, Spring Valley, CA, USA) was followed, and 2 × 25 μL serum was analyzed for the estradiol level (pg/mL) according to the manufacturer’s instructions. The data, given as estradiol levels (pg/mL), were shown as means and analyzed using Student’s two-tailed, unpaired *t*-test, see Supplementary Table 5. Notably, our data were comparable to previous findings^31^.

#### High performance liquid chromatography (HPLC) and electrochemical detection of monoamines and GABA

After decapitation, the brain was quickly removed from the skull and different brain regions were rapidly dissected, placed on dry ice, weighed (mg), and stored at −80 °C. At the day of HPLC analysis, the brain tissue samples were briefly sonicated in Eppendorf vials containing 200–800 μL (about 1:20 w/v) of 0.1 M perchloric acid (PCA) with antioxidants (0.2 g/L Na_2_S_2_O_5_, 0.05 g/L Na_2_-EDTA) and centrifuged at 20 627 × g for 20 min at 4 °C. The supernatant was used for HPLC analysis of monoamines and gamma-aminobutyric acid (GABA). The perchlorate extracts were immediately used for analysis of monoamines and stored at 4 °C for maximally 2 weeks until analysis of GABA. Levels (pmoles/mg wet tissue) of noradrenalin (NA), dopamine (DA), serotonin (5-hydroxytryptamine, 5-HT) were assessed by reverse-phase HPLC with electrochemical detection, essentially as described previously^32^, but using a mobile phase consisting of 10% methanol (v/v), 20 g/L citric acid monohydrate, 100 mg/L octane-1-sulfonic acid sodium salt, 40 mg/L EDTA dissolved in Milli-Q water and pH adjusted to 4.0 according to the protocol (Santiago *et al.* 2010). The Merck-Hitachi HPLC system consisted of an L-7100 pump, an L-7200 autosampler, a D-7000 interface and an electrochemical detector with in-built column oven (Decade, Antec, Leiden, The Netherlands), connected to a computer equipped with D-7000 version 2.0 chromatography software. The mobile phase was pumped at a flow rate of 0.9 mL/min through a Waters Spherisorb S5 ODS2 guard column (4.6 × 30 mm, Waters Corp, Milford, MA, USA) and a Waters Spherisorb S3 ODS2 cartridge analytical column (4.6 × 150 mm, Waters Corp). A mixture of external standards of monoamines and its metabolites (Sigma-Aldrich Co) was injected (0.5–1 pmol per compound) to identify and quantify the levels of NA, DA and 5-HT in the tissue samples. Before analysis of the level of GABA (nmoles/mg wet tissue) using o-phthalaldehyde (OPA)–sulphite derivatization and electrochemical detection according to the protocol^33^, the perchlorate extract (0.1 M PCA) was neutralized with NaOH (5.0 M) (~3 μL in 100 μL) and diluted 250 × or 500 × with milliQ water. A Shimadzu autosampler (SIL-10 AF, Shimadzu Corp, Kyoto, Japan) was set to add 2 μL OPA-sulphite solution to 100 μL diluted sample and after 1 min 5–20 μL of the derivatized sample was injected into the HPLC system. The same type of guard and analytical column as described above for monoamines was used. The mobile phase consisted of 0.1 M monosodium phosphate buffer and 0.5 mM EDTA with 25% methanol (v/v) water adjusted to pH 4.5 with 1 M phosphoric acid, and the OPA-sulphite solution consisted of 22 mg OPA (Sigma-Aldrich Co), dissolved in 0.5 mL sodium sulphite (1 M) to which was added 0.5 mL of absolute ethanol and 0.9 mL of sodium tetraborate buffer (0.1 M) adjusted to pH 10.4 with 5 M sodium hydroxide (this solution remained stable for 2 days when stored in a dark vial at 4 °C). Flow rate was 0.9 mL/min and the electrochemical cell was set to 850 mV vs. Ag/AgCl. An external GABA standard (4–8 pmoles) was injected prior to the tissue samples. Under our conditions, GABA eluted at 4.41 min. Data from male and female mice were comparable (confirmed by one-way ANOVA with Tukey’s *post hoc* comparison test (data sets with same variance (Bartlett’s test) or Kruskal-Wallis with Dunn’s *post hoc* comparison test (data with unequal variance (Bartlett’s test)) and subsequently pooled. The pooled data, given as pmoles/mg wet tissue (DA, NA, 5-HT) or nmoles/mg wet tissue (GABA), were shown as means ± s.e.m., and analyzed using Student’s two-tailed, unpaired *t*-test with Welch’s *post hoc* correction where indicated in Supplementary Table 6.

#### Glutamate assay

The mice were decapitated and the brains were quickly removed and dissected. CTX, HC and CRBL from 5 mice were pooled and lysates were prepared according to the glutamate assay protocol (Glutamate Colorimetric Assay Kit K629-100, BioVision Inc, Milpitas, CA, USA). The protein content in lysates was determined (Bio-Rad Protein Assay Kit II 500-002, Bio-Rad Laboratories Inc, Hercules, CA, USA), and 4 × 20 μg protein was analyzed for the glutamate content according to the manufacturer’s instructions. The data, given as glutamate levels (nmoles/20 μg protein), were shown as means ± s.e.m. and analyzed using Student’s two-tailed, unpaired *t*-test with Welch’s *post hoc* correction where indicated in Supplementary Table 5.

#### Primary mouse cultures

Primary hippocampal mixed astrocyte and neuron cultures of all genotypes were established from E17 embryonic mice brains as described previously^34^. The mixed cultures were seeded at a density of 0.5 × 10^5^ cells/cm^2^ and grown in supplemented Neurobasal media (Neurobasal 21203, 1 μM L-glutamine, B-27 supplement, 50 μg/mL penicillin-streptomycin; all reagents were from GIBCO Laboratories). The mixed astrocyte neuron cultures were propagated for 20–25 days, to ensure maturation of the astrocytes. Twice a week, 50% of the media covering the mixed cultures were replaced. The primary mixed cultures (day 21) were validated by immunocytochemistry (data not shown): Culture-covered cover slips were washed 2 × with warm PBS (7.7 mM Na_2_HPO_4_, 2.3 mM NaH_2_PO_4_, 150 mM NaCl), and the cells were subsequently fixed to the cover slip (4% paraformaldehyde, for 5 min at 4 °C), and washed 2 × in PBS. The cells were permeabilized (0.1% Triton X-100, for 3 min at RT), and washed (1 hour at RT) in PBS with 10% bovine serum albumin (BSA) (Sigma-Aldrich Co). Cover slips were incubated with primary antibodies diluted in PBS with 5% BSA (2 hours at RT): Polyclonal goat anti-human glial fibrillary acidic protein (GFAP) IgG (C-terminus of GFAP, sc-6170 (C-19), Santa Cruz Biotechnology Inc) diluted 1:200, and mouse anti-Pan Neuronal Marker protein IgG (blended monoclonal antibody cocktail, MAB2300, Millipore Corp) diluted 1:1 000. After antibody incubation, the coverslips were washed 3 × in PBS and incubated with secondary antibodies diluted in PBS with 5% BSA (1 hour at RT): Alexa 488-conjugated donkey anti-goat antibody (1:2 000, A11055, Invitrogen Corp, Carlsbad, CA, USA) and Cy3-conjugated donkey anti-mouse antibody (1:2 000, 715-165-150, Jackson ImmunoResearch Laboratories Inc, West Grove, PA, USA). After 3 × PBS wash, the cover slips were mounted with ProLong Gold antifade reagent with DAPI (P36931, Invitrogen Corp) which stains nuclei of both astrocytes and neurons. Fluorescence microscopy examination of the stained cover slips revealed that mixed cultures were comparable to the cultures obtained by others^34^ (not shown), with a 1:1 ratio of astrocytes and neurons. The specificity of the GFAP antibody was previously confirmed on formalin-fixed cerebellar cell primary culture and allowed for staining of astrocyte processes and cell bodies (company product sheet), and the specificity of the Pan Neuronal Marker antibody was previously confirmed on rat E18 cortex primary cells and allowed for staining of axons, dendrites, nucleus and cell body of neurons (company product sheet). The primary mixed cultures (day 21) were validated by SDS-page Western blotting: 100 ug of each lysate were separated by SDS-page and Western blotted afterwards essentially as described above. The Western blot was divided in two, and the level of α_2_-protein (part >50 kDa) and the control protein, actin (part <50 kDa), was investigated using primary antibodies ((polyclonal rabbit anti-human α_2_ aa 432–455, 07674 Upstate, Millipore Corp) or (monoclonal mouse anti-chicken gizzard muscle actin, 612656, BD Transduction Laboratories, Franklin Lakes, NJ, USA)) and incubated over-night at 4 °C in dilution 1: 3 000 in PBS-T with 3% BSA. The blot parts were incubated with secondary antibodies ((HRP-conjugated swine anti-rabbit immunoglobulins, P0399, Dako Inc) or (HRP-conjugated sheep anti-mouse IgG, NXA931, GE Healthcare)) 2 hours at RT diluted 1:3 000 in PBS-T with 5% nonfat dry milk. The blots were developed and visualized as described above. The intensity of the bands in question was quantified as arbitrary units, AU. Densitometry was performed using the ImageJ software (Rasband, W.S., ImageJ, U. S. National Institutes of Health, Bethesda, Maryland, USA, http://imagej.nih.gov/ij/, 1997–2014.), see Supplementary Table 5. The specificity of the actin antibody was confirmed on a Western blot of Jurkat cell lysate and allowed for recognition of a protein of 42 kDa (company product sheet).

#### [^3^H]-D-aspartate uptake

On day 21–25, primary mixed cultures (propagated on 18-mm-cover slips in 12-well-plates) were washed 3 × with 0.5 mL Hepes Krebs-Ringer buffer. Aspartate uptake was allowed for 5 min at RT by incubation in 0.5 mL PBS containing 200 μM D-aspartate and [^3^H]-D-aspartate 0.15 μCi/well (PerkinElmer Inc, Waltham, MA, USA). Cover slips were rinsed 3 × with 0.5 mL cold Hepes Krebs-Ringer buffer, and subsequently lysed in 0.5 mL 0.1 M NaOH. The protein content in all lysates was determined using a method adapted from Bradford^25^ (RC DC protein assay kits, 500-0113 and 500-0114, Bio-Rad Laboratories Inc). The content of [^3^H]-D-aspartate in 0.45 mL lysate was mixed with 5 mL of scintillator (Ready Safe, 141349, Beckman Coulter Inc, Brea, CA, USA) and quantified by liquid scintillation spectrometry using a WinSpectral 1414 scintillation counter (Wallac, Turku, Finland). The counts per minute (CPM) were normalized to the protein amount (μg), see Supplementary Table 5. In each experiment, the average [^3^H]-D-aspartate uptake (CPM/μg protein) for WT samples was set to 100% and, subsequently, all samples were normalized (in %) relative to WT. The normalized data ([^3^H]-D-aspartate uptake as % of WT) were shown as means ± s.e.m. and analyzed using one-way ANOVA, see Supplementary Table 5.

#### Acoustic startle response

The acoustic startle reflex is a stereotyped motor response evoked by sudden, intense acoustic stimuli and characterized by contractions of the major muscles of the body, primarily mediated by a relatively simple, oligosynaptic pathway, located in the lower BS, that activates spinal and cranial motor neurons^35^. The acoustic startle response protocol^36^ was modified and implemented to the StartFear Combined system setup (Panlab Harvard Apparatus) and the accompanying software was used for data acquisition. The mouse was placed in a Plexiglas cylinder in the test chamber. The restrained mouse was firstly habituated to the apparatus for 5 min and then exposed to 6 series each consisting of 7 sound pulses (none, 80, 90, 100, 105, 110, 120 dB), presented in a random order with an inter-trial interval of 30 s. Movements within the cylinder were detected and transduced by a piezoelectric accelerometer attached to the Plexiglas base. In all trials, except null trials in which there was only background noise (none ~45 dB (mini sound level meter 325, Center Technology Corp, New Taipei City, Taiwan)), the pulse was 40 ms. The acoustic startle setup was cleaned and wiped with paper towels between each trial. Data from male and female mice were comparable (confirmed by two-way ANOVA) and subsequently pooled. The pooled data, shown as acoustic startle responses (%) at the indicated sound pulses (dB), were given as means ± s.e.m., and analyzed using two-way ANOVA and Student’s two-tailed, unpaired *t*-test, see Supplementary Table 4.

#### Marble burying

Increased marble-burying behavior is well-recognized as a mouse trait that mimics the compulsive behavior of OCD^37^. The marble burying protocol^37^ was followed. A mouse was placed in a clear plastic box (42 × 26 × 18 cm) containing 25 glass marbles (1.5-cm-diameter) evenly spaced upon a 5 cm deep layer of bedding (Tapvei 4HV aspen bedding, Tapvei Ltd, Korteinen, Finland), without food and water. After 30 min, the number of marbles buried to at least two-thirds of the depth was counted. The burying was documented by photography (Canon Digital IXUS 970 IS camera, Canon, Ōita, Japan). The marbles were cleaned with 3 × water and wiped with paper towels between each trial, and a clean plastic box with bedding was used per mouse. The data, given as marbles buried (n), were shown as means ± s.e.m. and analyzed using Student’s two-tailed, unpaired *t*-test and one-way ANOVA with Tukey’s *post hoc* comparison test, see Supplementary Table 4. Notably, female and male WT mice buried marbles at levels previously observed for a mouse strain with a related background^38^.

#### Elevated plus maze

Mice normally display an aversion of open spaces, which involves avoidance of open areas by confining movements to enclosed spaces or to the edges of a bounded space, and in elevated plus maze (EPM) this translates into preference of movement to the enclosed arms^39^. The EPM is a plus-shaped maze that is elevated 50 cm above the floor (Stoelting Co, Wood Dale, IL, USA). It consists of two opposite enclosed arms surrounded by 15-cm high opaque walls and two open arms of the same size (5 × 35 cm). The maze was set up in a dim lit room under a video camera (Panasonic WV-BP332 Surveillance camera, Panasonic Corp, Tokyo, Japan) connected to a computer under the control of the ANY-maze tracking system. Each mouse was placed in the center (5 × 5 cm) of the maze facing one of the open arms. Testing sessions of 10 min were carried out for each mouse and measured the distance (cm) and the number of entries and the time spent in the open arms. The maze was cleaned with 70% ethanol and wiped with paper towels between each trial. The percentage of entries into the open arms was calculated as follows: (open arm entries (n)/(open arm entries (n) + closed arm entries (n))) × 100, and percentage of time (s) spent in the open arms was calculated as follows: ((total time (s) in open arms)/(600 s)) × 100. The experiment was repeated implementing a stress-protocol where the mouse was placed 30 min on a 20 × 20 cm transparent platform raised 1 m above the floor prior to the Elevated plus maze experiment^40^. Data from male and female mice were comparable (confirmed by one-way ANOVA with Tukey’s *post hoc* comparison test (data sets with same variance (Bartlett’s test) or Kruskal-Wallis with Dunn’s *post hoc* comparison test (data with unequal variance (Bartlett’s test)) and subsequently pooled. The pooled data, given as distance (cm), open arm time (%), open arm entries (%), and line crossings (n), were shown as means ± s.e.m. and analyzed using Student’s two-tailed, unpaired *t*-test with Welch’s *post hoc* correction where indicated in Supplementary Table 4.

#### Three-chamber social preference

The three-chamber apparatus is a Plexiglas rectangle with two transparent partitions that make left, center (center, C), and right chambers (each chamber is 20 × 43 cm) (Stoelting Co). Each partition has a square opening (8 × 5 cm) in the bottom center, moreover, the left and right chamber each have a 5 cylindrical wire cage (7-cm-diameter) which was used as an inanimate object or the cage housing a stranger mouse. The three-chamber unit and wire cups were cleaned with 70% ethanol and wiped with paper towels between each trial. The mouse was placed in the center of the three-chamber unit and after 5 min of habituation, the doors were opened. In the next 10-min session, the mouse was allowed access to all three chambers, and in one of the chambers an age- and gender-matched C57BL/6 J mouse (stranger, S) that had never been exposed to the mouse being tested, was placed in one of the two wire cages. The wire cage on the other side remained empty (empty, E). The movement of the mouse was recorded by a Panasonic WV-BP332 Surveillance camera (Panasonic Corp) connected to a computer under the control of the ANY-maze tracking system. Time spent in each chamber (chamber time (s)), entries into each chamber (chamber entries (n)), and time spent within a ~2 cm zone proximal to each wire cage (sniffing time (s)) were measured. The data, given as chamber time (s), sniffing time (s) and chamber entries (n), were shown as means ± s.e.m. and analyzed using one-way ANOVA with Tukey’s *post hoc* analysis, see Supplementary Table 4. Notably, female and male C57BL/6J mice were social, which was in agreement with previous findings^41^.

#### Tail suspension

Mice with a stress-induced depression-phenotype are immobile for a longer time period compared to controls when tested in the tail suspension test (TST), and they will terminate their escape-like behaviors earlier than controls leading to less movement of the body (detected by absolute turn angles). We used the tail suspension test (TST)^42^ to test the α_2_^+/G301R^ mice for a stress-induced depression-like phenotype and the tail suspension test protocol^43^ was followed. The mouse was suspended by the tail using adhesive tape to a horizontal bar (mouse-tip was raised 60 cm from the ground). The mouse was recorded (Canon Digital IXUS 970 IS camera, Canon) for 6 min and mice that climbed their tail were excluded from the experiment (in total four WT mice and one α_2_^+/G301R^ mouse were omitted due to tail climbing). The majority of the films were examined manually (all blindly), and the full immobility time (s) per mouse was determined by use of a stopwatch (data not shown). Additionally, all the films were analyzed using the ANY-maze tracking system. Alignment between the manual examination and the ANY-maze recordings were ensured and “88% immobility” in the ANY-maze settings generated an average difference of 6.8% between manual immobility time (s) examination and ANY-maze tracking of immobility time (s), n = 33 mice (comparison not shown). Moreover, the ANY-maze tracking system was used to examine the absolute turn angles (degrees) per mouse, the number of rotations (n) and the mobility time (s). The ANY-maze-generated data were presented in three time intervals on the x-axis adding to 6 min in total. Data from male and female mice were comparable (confirmed by one-way ANOVA with Tukey’s *post hoc* comparison test) and subsequently pooled. The pooled data, given as immobility (s), mobility (s), absolute turn angles (degrees), and number of rotations (n) in the given time intervals, were shown as means ± s.e.m. and analyzed using two-way ANOVA, see Supplementary Table 4. All movies were manually and blindly examined for audible squeaks, and if a mouse squeaked it was denoted 100% whereas a silent mouse was denoted 0%. Data from male and female mice were comparable (confirmed by one-way ANOVA with Tukey’s *post hoc* comparison test) and subsequently pooled. The pooled data, given as vocalization (%), were shown as means ± s.e.m. and analyzed using Student’s two-tailed, unpaired *t*-test, see Supplementary Table 4.

#### Stress-induced sucrose preference

The stress-induced sucrose-preference test models the specific depression-like syndrome denoted anhedonia^44^. In the stress-induced sucrose-preference test, normal mice experience pleasure from drinking a sweet solution, and reduced preference for a sweet solution over pure water represents an anhedonic phenotype (= decreased ability to experience pleasure). The stress-induced sucrose preference protocol^44^ was modified. Mice were housed with littermates of same genotype and sex. No previous food or water deprivation was applied before the experiment. Every cage had access to a bottle with pure water and a bottle with 2% sucrose solution (1.07687.1000, Merck KGaA, Darmstadt, Germany) which were weighed every day (and the exact time point was noted), and the placing of the bottles were swapped to prevent possible effects of side preference in drinking behavior. Before the experiment, the mice were habituated to the bottles for 3 days, and hereafter consumption was measured by weighing of the bottles. Weighing data in grams (g) were collected for each bottle in every cage over a period of 3 days (none), 2 days after completion of the first stress protocol (Stress I) and 2 days after completion of the second stress protocol (Stress II)). The first stress protocol^45^ involved 3 × foot shock (0.7 mA for 1 s) (Stress I). The second stress protocol involved a series of experiments distributed over a period of 11 days (Stress II): 2 × tail suspension tests for 6 min (see protocol above), 3 × fixations (inhibitory stress) using stress cones (mouse decapicone disposable mouse restrainers DC M200, Braintree Scientific Inc, Braintree, MA, USA) for 60 min, and 3 × forced swim tests (the mouse was released into a transparent cylinder (1 L beaker, Ø 10 cm) filled with 30 °C warm water with a depth of 13 cm for 6 min (the water was regularly changed between subjects). For every day, the percentage of sucrose intake was calculated as follows: (sucrose intake (g)/(sucrose intake (g) + water intake (g))) × 100, and the liquid intake given as: ((2% sucrose intake (g) + water intake (g)) per day/(n) mice in the given cage). For every cage, the average cage-intake of sucrose (%) and liquid consumption (g/mouse/day) over 3 days (None), and 2 days (Stress I and Stress II) were determined. Data from male and female mice were comparable (confirmed by one-way ANOVA with Tukey’s *post hoc* comparison test) and subsequently pooled. The pooled data, given as 2% sucrose intake relative to water intake (%) and liquid intake (g/mouse/day) at the given protocols, were shown as means ± s.e.m. and analyzed using Student’s two-tailed, unpaired *t*-test with Welch’s *post hoc* correction where indicated in Supplementary Table 4. Notably, the reduced sucrose preference in a subset of WT mice after a stress protocol is in agreement with previous findings^46^.

### Drug administration

Amantadine (A1260-5G, Sigma-Aldrich Co) dissolved in solvent (10 mg/mL, PBS (pH 7.4)) was administered by an i.p. injection in a volume of 30 mg/kg 30 min prior to behavioral testing. Memantine hydrochloride (M9292, Sigma-Aldrich Co) dissolved in solvent (0.6 mg/ml, PBS (pH 7.4) was administrated by an i.p. injection of 3 mg/kg 30 min prior to behavioral testing.

Depoprovera^®^ (50 mg/mL, Vnr 01 70 20, Pfizer ApS, Puurs, Belgium) diluted 2.5 × in PBS (pH 7.4) was administered by a subcutaneous (s.c.) injection in the neck in a volume of 100 μL resulting in 2 mg Depoprovera/mouse ~3 weeks before (behavioral) experiments.

### Bioinformatics

The UCSC genome browser^47^ was used to examine the human Feb. 2009 (GRCh37/hg19) assembly. The microsatellite D1S1677 (UCSC STS id: 5 370) was located to chromosome 1, band q23.3 (163 559 700-165 360 041) and the full length *ATP1A2-*gene is located on chromosome 1, band q23.2 (160 085 520-160 113 374) (uc001fvc.3). The distance from microsatellite marker D1S1677 to the *ATP1A2-*gene was in the range 3 446 326-5 274 521 bp’s which corresponds to 3.4-5.3 cM (under the assumption that 1 cM is equivalent, on average, to 1 million bases in the human genome).

### Statistical analysis

All data were shown as mean ±s.e.m. and statistical analyses were done using Prism version 5.01 or 6.03 (GraphPad Software Inc, La Jolla, CA, USA or the R software (R Foundation for Statistical Computing, Vienna, Austria))^48^. The obtained male and female mice data were only pooled when this was statistically validated (*P* < 0.05), and all statistical tests and the *P*-values obtained are presented in Supplementary Table 4. The Welch’s *post hoc* correction was used when data sets with unequal variances were analyzed using the unpaired two-tailed *t*-test. The Tukey’s *post hoc* comparison test was used when data sets with the same variances were analyzed using the one-way ANOVA analysis, and the Kruskal-Wallis with Dunn’s *post hoc* comparison test was used when data sets with unequal variances were analyzed using the one-way ANOVA analysis. The threshold value (alpha) was set to 0.05.

## Results

### Generation and basic characterization of the α_2_^+/G301R^ knock-in (KI) mouse

The G→A mutation in exon 8 encoding the FHM2-associated G301R-mutation was introduced by homologous recombination and resulted in α_2_^+/G301R^ knock-in (KI) mice (Supplementary Table 1 and Supplementary Fig. 1a). Several analyses confirmed successful gene targeting and Cre-recombinase-treatment (Supplementary Fig. 1a–d,f). The distribution of sex and genotypes among offspring after breeding of C57BL/6 J mice with α_2_^+/G301R^ mice (backcrossed to N ≥ 8) was according to Mendel’s law of segregation (Materials and Methods), and the α_2_^+/G301R^ mice appeared normal and indistinguishable from WT mice by eye. Crossing of two α_2_^+/G301R^ mice generated homozygous (−/−) α_2_^G301R/G301R^, heterozygous (+/−) α_2_^+/G301R^, and WT (+/+) α_2_^+/+^ pups, which were identified by genotyping PCR (Supplementary Fig. 1e). The α_2_^G301R/G301R^ pups died immediately after birth resembling the neonatally lethal phenotype previously reported for another mouse model targeting the *Atp1a2*-gene^23^. The design of all behavioral tests allowed for detection of potential gender-coupled differences, and the obtained male and female mice data were only pooled when this was statistically validated (*P* < 0.05), see Supplementary Table 1. Moreover, all behavioral experiments were done blind to genotype with age-matched littermates. Additionally, since ovarian hormones can modulate migraine, they may also be able to influence FHM2-related phenotypes, and therefore, we conducted our assay, if applicable, to allow discrimination of female and male phenotypes.

Followed for a year, the body weight progression of male α_2_^+/G301R^ mice was different from that of WT mice in a period of 4 months (interval: 60–190 days), whereas the body weight progression of female α_2_^+/G301R^ mice was similar to that of WT mice (Supplementary Fig. 1 g), showing that obesity was not a trait of female α_2_^+/G301R^ mice.

Western blotting analysis revealed reduced α_2_ protein levels in brain lysates from embryonic (E17.5) and newborn (day 0) α_2_^G301R/G301R^ pups, and they harbored 71% and 94% reduced α_2_ protein levels, respectively, compared to WT pups (Supplementary Fig. 2a,b). Brain lysates from embryonic and newborn α_2_^+/G301R^ pups also showed 33–41% reduced α_2_ protein levels compared to WT pups (Supplementary Fig. 2a). Moreover, hippocampal-derived *in vitro* cultures established from embryos (E17) of all genotypes harbored 99% reduced (α_2_^G301R/G301R^) and 77% reduced (α_2_^+/G301R^) α_2_ protein levels compared to WT (Supplementary Fig. 2c and Supplementary Table 2). For adult α_2_^+/G301R^ mice, reduced α_2_ protein levels were found in all male brain areas (36–78%) including hippocampus (HC), cerebellum (CRBL), cortex (CTX), and brain stem (BS) compared to WT mice (Supplementary Fig. 1b), largely confirmed by parallel analyses of adult female brain lysates (Supplementary Fig. 1 h and Supplementary Table 2).

Parallel investigation of the levels of the brain-expressed α-isoforms, α_1_, α_2_ and α_3_, in various brain lysates from male and female α_2_^+/G301R^ and WT mice showed that comparable levels of α_1_ and α_3_ isoforms were observed in all brain regions (Supplementary Fig. 2d, for quantifications see Supplementary Fig. 2e and Supplementary Table 2). Moreover, reduced amounts of the α_2_ isoform were observed in all brain lysates from α_2_^+/G301R^ mice (female and male) compared to WT littermates (Supplementary Fig. 2d, for quantification see Supplementary Fig. 2e and Supplementary Table 2).

### Recovery phase after cortical spreading depression is prolonged in male α_2_^+/G301R^ mice

The propagation speed of CSD was measured between two recording electrodes during application of potassium acetate (KAc) (Fig. 1a). No differences in the propagation speed of a single CSD event when comparing male α_2_^v/G301R^ and WT mice were observed (data not shown).

CSD induced a reduction in the ECoG signal in both WT and α_2_^+/G301R^ mice (Fig. 1b). After CSD, both the depolarization and the disturbances in the brain ion homeostasis regenerate within minutes in the normal brain, and *in vivo* ECoG results showed that regeneration after CSD-induction was significantly reduced in brains from male α_2_^+/G301R^ mice, compared to WT mice (Fig. 1c,d). Moreover, the evoked potentials had recovered to approx. 87% of *pre*-CSD values 15 min after CSD (see Supplementary Table 3), and no difference was observed between the genotypes. This is similar to what have been shown in two FHM1 knock-in mouse models^49^, suggesting that the spontaneous activity (ECoG signal) is more likely affected in α_2_^+/G301R^ mice than the evoked activity.

### Cerebral blood flow in male α_2_^+/G301R^ mice is comparable to WT after cortical spreading depression induction

Human brain scans have shown cerebral blood flow (CBF) changes in migraineurs after CSD, denoted spreading oligemia^15^. Laser-Doppler flowmetry showed that CBF in male α_2_^+/G301R^ mice was comparable to CBF in WT mice after CSD induction in the timespan (1 hour) investigated (Fig. 1e,f).

### Hypoactivity in female α_2_^+/G301R^ mice is attributed to their sex hormone cycle

**Figure 2:**
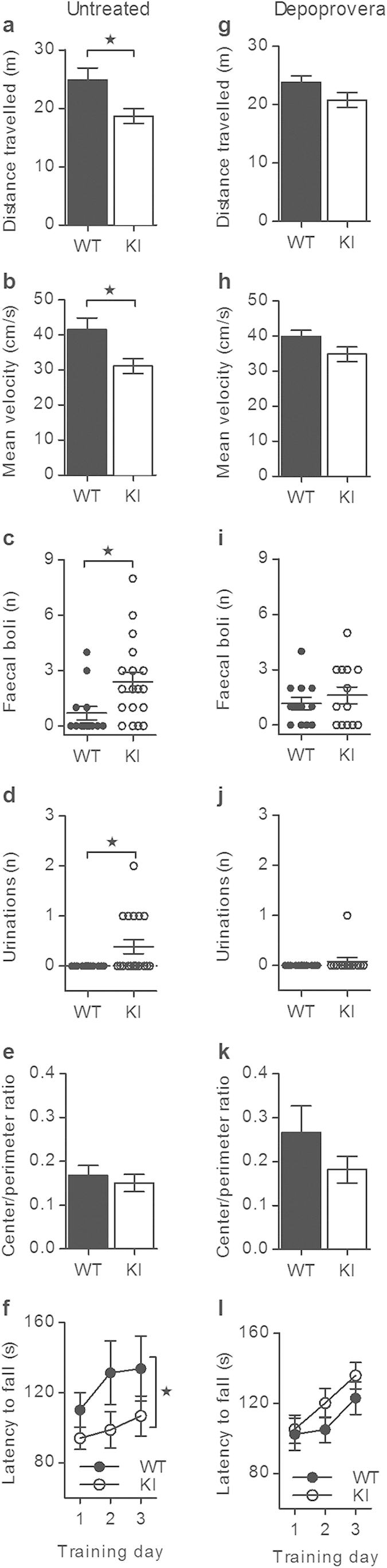
Hypolocomotion and reduced motor skill learning/coordination of female α_2_^+/G301R^ mice were rescued with Depoprovera. (**a,b**) Female α_2_^+/G301R^ mice (n = 18) travelled a shorter distance (m) (**a**), and moved with a reduced mean velocity (cm/s) (**b**) compared to WT (n = 13). (**c,d**) Female α_2_^+/G301R^ mice (n = 18) produced more droppings (n) (**c**) and urinations (n) (**d**) compared to WT mice (n = 13). (**e**) The center/perimeter ratios were the same for female α_2_^+/G301R^ (n = 18) and WT mice (n = 13). (**f**) Female α_2_^+/G301R^ mice (n = 5) had a decreased latency to fall (s) from the rotarod compared to WT mice (n = 4). (**g–j,l)** Depoprovera-treated female α_2_^+/G301R^ (n = 13) and WT (n = 12) mice travelled the same distance (m) (**g**), moved with the same mean velocity (cm/s) **(h**), had equal dropping (n) (**i**) and urination (n) (**j**) behaviors, and female α_2_^+/G301R^ mice (n = 9) gained comparable latencies to fall (**s**) from the rotarod to WT mice (n = 8) (**l**). (**k**) The center/perimeter ratios of Depoprovera-treated female α_2_^+/G301R^ (n = 13) and WT mice (n = 12) were the same. Data shown as means ± s.e.m. ★*P* < 0.05. (See also Supplementary Figs 3–5).

Open field and rotarod tests revealed that only female α_2_^+/G301R^ mice exhibited behaviors different from WT mice (Fig. 2a–d,f), where male α_2_^+/G301R^ and WT mice were indistinguishable from each other in open field experiments (Supplementary Fig. 3). Hypolocomotion was observed in female α_2_^+/G301R^ mice as they travelled a shorter distance and moved more slowly than WT mice (Fig. 2a,b), and female α_2_^+/G301R^ mice were also more distressed (increased dropping and urination behaviors) compared to WT mice (Fig. 2c,d). Agoraphobia was not a trait since the center/perimeter ratios for female α_2_^+/G301R^ and WT mice were comparable (Fig. 2e). Endurance, learning and coordination were decreased in female α_2_^+/G301R^ mice, as they spent less time on the accelerated rotarod compared to WT mice (Fig. 2f). Grip strength was comparable for α_2_^+/G301R^ and WT mice (Supplementary Fig. 4) reinforcing that α_2_^+/G301R^ mice had normal neuromuscular functions.

Depoprovera is a progestin-only contraceptive that suppresses the natural cyclic fluctuations of female sex hormones by ensuring a low and stable estradiol level^50^, and the efficiency of a single subcutaneous injection of Depoprovera on the serum estradiol level in WT mice was confirmed (Supplementary Fig. 5). Interestingly, Depoprovera-treated female α_2_^+/G301R^ mice were indistinguishable from Depoprovera-treated WT mice in all open field and rotarod tests, showing that the specific behaviors observed for the untreated female α_2_^+/G301R^ mice were rescued to WT behaviors (Fig. 2g–j,l).

### The α_2_G301R KI mutation impaired glutamate transport

High performance liquid chromatography (HPLC) analyses of lysates (frontal CTX, occipital CTX, striatum (STR), HC, BS, and CRBL) from α_2_^+/G301R^ and WT mice showed comparable levels of dopamine (DA), noradrenaline (NA), serotonin (5-HT), and gamma-aminobutyric acid (GABA) (Supplementary Fig. 6).

**Figure 3:**
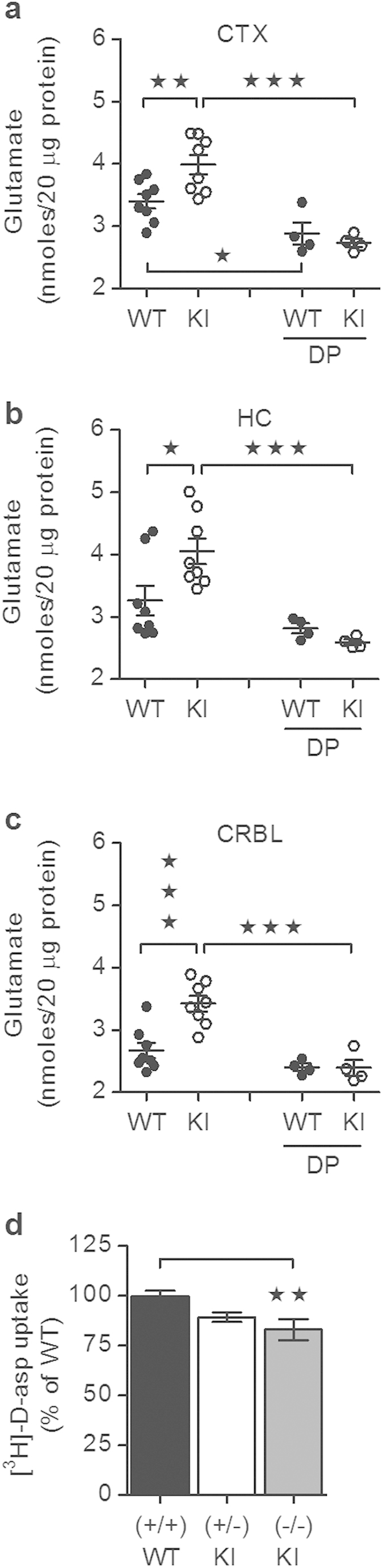
Glutamate uptake function and glutamate levels in various female brain areas were affected by G301R Mutation(s). (**a–c**) Glutamate levels (nmoles/20 μg protein) were determined in lysates from cerebral cortex (CTX) (**a**), hippocampus (HC) (**b**) and cerebellum (CRBL) (**c**) of untreated and Depoprovera (DP)-treated female α_2_^+/G301R^ (n = 2 × 5 and n = 1 × 5, respectively) and WT (n = 2 × 5 and n = 1 × 5, respectively) mice. (**d**) Glutamate uptake function, evaluated as [^3^H]-D-aspartate uptake (and given as uptake relative to WT uptake (in %)), was reduced in hippocampal-derived *in vitro*-matured mixed cultures of neurons and astrocytes from α_2_^G301R/G301R^ (−/−) (n = 12) compared to WT (+/+) (n = 20), but largely similar to WT in culture from α_2_^+/G301R^ (+/−) (n = 22). Data shown as means ± s.e.m. ★*P* < 0.05, ★★*P* < 0.01, ★★★*P* < 0.001. (See also Supplementary Fig. 6).

Elevated glutamate levels (calorimetrically assayed) were observed in CTX (17%), HC (24%) and CRBL (28%) lysates from female α_2_^+/G301R^ mice compared to lysates from WT mice (Fig. 3a–c). Furthermore, Depoprovera treatment reduced the glutamate levels in CTX, HC and CRBL lysates from female α_2_^+/G301R^ mice to levels comparable to those from WT mice (Fig. 3a–c). In HC and CRBL lysates from Depoprovera-treated WT mice, the glutamate levels were indistinguishable from those from untreated WT mice (Fig. 3b,c). However, in CTX lysates from WT mice, the glutamate level was slightly reduced by the Depoprovera treatment (Fig. 3a).

The glutamate transport, measured as [^3^H]-D-aspartate uptake function, in hippocampus-derived *in vitro* embryo primary cultures derived from α_2_^+/G301R^ and α_2_^G301R/G301R^ embryos, revealed that glutamate uptake function was impaired in cultures from α_2_^G301R/G301R^ E17 embryonic mice compared to WT (Fig. 3d).

### The α_2_^+/G301R^ mice showed increased responsivity to acoustic stimuli attributed to glutamate system defects

**Figure 4:**
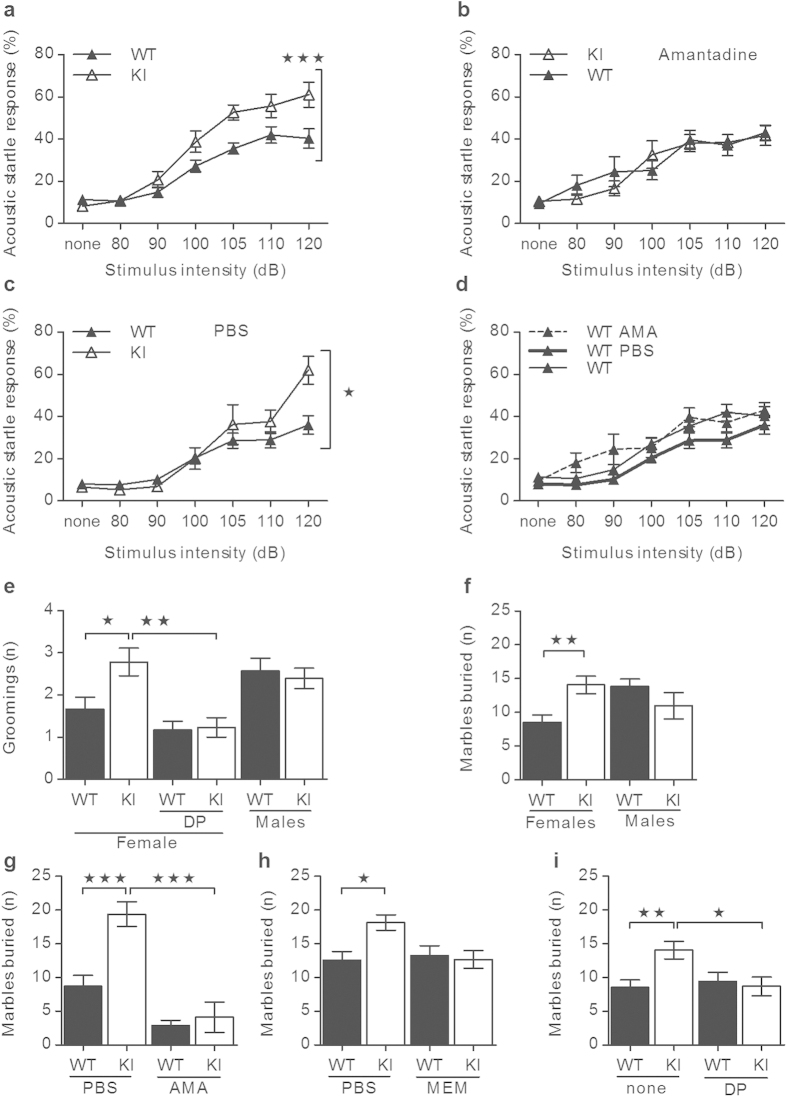
Increased acoustic startle response and compulsive behaviors in α_2_^+/G301R^ mice were rescued with amantadine (females and males), memantine or Depoprovera (females). (**a,b**) Increased acoustic startle response (ASR) (%) was observed for α_2_^+/G301R^ mice (n = 7–9) compared to WT (n = 7–8) **(a**) and the ASR was rescued by amantadine (AMA) treatment (**b**). (**c,d**) Increased ASR (%) was observed for PBS-treated α_2_^+/G301R^ mice (n = 5) compared to WT (n = 12) (**c**). ASR (%) of untreated (none) WT mice (n = 8), and WT mice receiving amantadine (AMA) treatment (n = 7) or PBS treatment (n = 12) was the same (**d)**. (**e**) Female α_2_^+/G301R^ mice (n = 18) groom more than WT mice (n = 12), whereas male α_2_^+/G301R^ mice (n = 23) groom as WT (n = 21). The increased grooming behavior of female α_2_^+/G301R^ mice (n = 13) was rescued by Depoprovera (DP)-treatment, and thus comparable to WT mice (n = 12). (**f–i**) In the marble-burying test, female α_2_^+/G301R^ mice (n = 23) buried more marbles than WT mice (n = 25) while male α_2_^+/G301R^ mice (n = 14) buried marbles as for WT mice (n = 31) (**f**). AMA-treated (**g**), MEM-treated (**h**) and DP-treated (**i**) female α_2_^+/G301R^ mice (n = 9, n = 10 and n = 20, respectively) and WT mice (n = 11, n = 11 and n = 18, respectively) buried comparable numbers of marbles, whereas PBS-injected female α_2_^+/G301R^ mice (n = 8–10) buried more marbles than WT mice (n = 11–15) (**g,h**); the difference in marble-burying observed for untreated and PBS-injected female α_2_^+/G301R^ mice (**f,g,h**) is attributed the injection 30 min prior to the experiment. The marble-burying data for untreated female α_2_^+/G301R^ and WT mice were shown both in (**f,i**). Data shown as means ± s.e.m. ★*P* < 0.05, ★★*P* < 0.01, ★★★*P* < 0.001.

Interestingly, both male and female α_2_^+/G301R^ mice responded more to the aversive acoustic stimuli compared to WT mice (Fig. 4a), and this prompted us to target the glutamate system using a weak antagonist (amantadine) of the NMDA type glutamate receptor. Amantadine-administration rescued the increased acoustic startle reflex (ASR) behavior in α_2_^+/G301R^ mice to that of WT mice (Fig. 4b). In parallel, sham treatment with solvent (phosphate-buffered saline (PBS)) had no influence on the ASR behavior in α_2_^+/G301R^ and WT mice (Fig. 4c), and, moreover, ASR behavior was the same in untreated (none) and amantadine (AMA)- or PBS-treated WT mice (Fig. 4d).

### Female α_2_^+/G301R^ mice mimicked more compulsive behaviors than male α_2_^+/G301R^ mice

For mice, pathological grooming behavior is considered to mimic human pathological grooming behaviors as e.g., hair pulling (trichotillomania), which frequently co-occurs with obsessive-compulsive spectrum disorders and OCD^51^. Male α_2_^+/G301R^ and WT mice displayed comparable levels of grooming behavior whereas female α_2_^+/G301R^ mice displayed an increased grooming level compared to WT mice (Fig. 4e). Depoprovera treatment reduced the grooming behavior of female α_2_^+/G301R^ mice to that of female WT mice, but had no effect on the grooming level of WT mice (Fig. 4e). The grooming level of female α_2_^+/G301R^ mice was comparable to the grooming level in male α_2_^+/G301R^ and male WT mice, and, the female α_2_^+/G301R^ mice had a healthy-looking coat without visible hair-less spots. Interestingly, increased facial grooming is described as a specific phenotype in a chronic rat model for migraine^52^, however, our data (Fig. 4e) cover both face and body grooming.

Increased marble-burying behavior of mice mimics compulsive-like behavior of OCD. Interestingly, only female α_2_^+/G301R^ mice displayed increased marble-burying behavior compared to WT mice (Fig. 4f), and both amantadine and memantine treatments rescued their behavior to that of WT mice (Fig. 4g,h). A subgroup (~30%) of normally cycling female rats displayed changes in marble-burying behavior along the estrous cycle, burying more marble during metestrous compared to proestrous of the menstrual cycle^53^, showing that marble-burying behavior is low in that menstrual phase where the estradiol level is low. Depoprovera treatment rescued the increased marble-burying behavior of female α_2_^+/G301R^ mice to that of WT mice (Fig. 4i), showing that this behavior was indeed influenced by the female sex hormones.

### Bioinformatics assessment does not exclude a genetic basis for OCD coupled to mutations in the *ATP1A2* gene

The *ATP1A2* gene is located on chromosome 1q23.2^6^. A genome-wide linkage scan showed a susceptibility locus for an early form of OCD on chromosome 1q with a logarithm of odds (LOD) score of +3 using microsatellite D1S1677^54^. We bioinformatically assessed these data, and found localization of microsatellite D1S1677 to the *ATP1A2* gene within the genetic distance range of 3.4–5.3 cM. Subsequently, a genome-wide association study did not identify any OCD genes^55^, and future targeted approaches are required to elucidate if *ATP1A2* is indeed an OCD-linked gene.

### α_2_^+/G301R^ mice are not anxious but display decreased sociability

The α_2_^+/G301R^ mice behaved as WT mice in the elevated plus maze (EPM) setup (Supplementary Fig. 7a–d). These data are in agreement with the lack of agoraphobic behavior in the open field test (Fig. 2e and Supplementary Fig. 3e). Despite implementation of a stress-protocol prior to the experiment, stressed α_2_^+/G301R^ and WT mice still displayed comparable behaviors in the EPM setup (Supplementary Fig. 7e–g).

**Figure 5:**
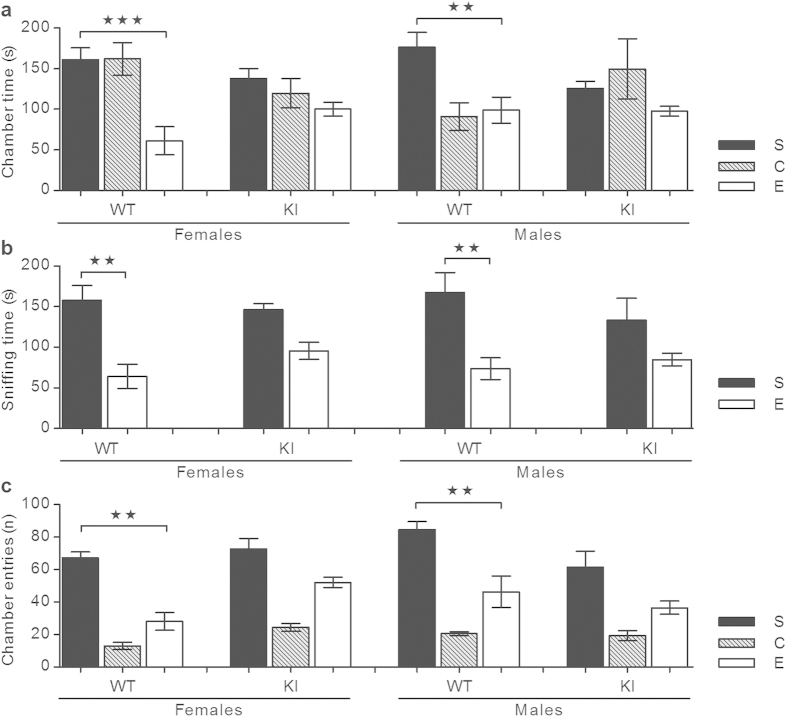
The α_2_^+/G301R^ mice displayed reduced sociability. (**a–c**) In a three-chamber sociability setup, WT mice (n = 5) spent more time (s) in the chamber housing a stranger mouse (S) than in the chamber housing the empty wire cage (E), whereas α_2_^+/G301R^ mice (n = 5) had no preference for either chamber (**a**). WT mice spent more sniffing time (s) around the stranger mouse (S) than the empty wire cage (E), whereas α_2_^+/G301R^ mice spent the same time (s) sniffing regardless of whether there was a stranger mouse (S) in the wire cage or not (E) (**b**). WT mice had more entries (n) into the chamber housing the stranger mouse (S) than the chamber with the empty wire cage (E), whereas α_2_^+/G301R^ mice had the same number of entries (n) into both chambers (**c**). C denoted the center chamber separating the two other chambers (E, S). Data shown as means ± s.e.m. ★★*P* < 0.01, ★★★*P* < 0.001.

A three-chamber sociability test showed that WT mice spend more time in the chamber containing the stranger mouse (S) than in the chamber containing the non-social/empty wire cage (E) (Fig. 5a). Moreover, WT mice spent more time sniffing (in close proximity to) the stranger mouse compared to sniffing the empty wire cage (Fig. 5b), and they made more entries into the chamber containing the stranger mouse compared to entries into the chamber containing the empty wire cage (Fig. 5c). Contrary to WT mice, the α_2_^+/G301R^ mice displayed a reduced sociability phenotype and spent comparable amounts of time in both chambers and comparable sniffing times to the stranger mouse and the empty wire cage (Fig. 5a,b), and they had the same number of entries into both chambers (Fig. 5c).

### α_2_^+/G301R^ mice suffer from stress-induced depression

**Figure 6:**
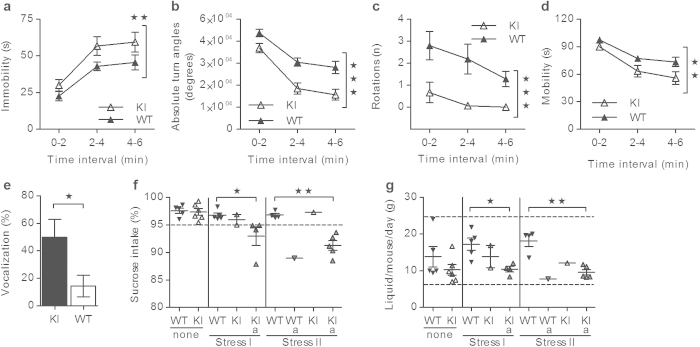
The α_2_^+/G301R^ mice displayed stress-induced depression. (**a–e**) The α_2_^+/G301R^ mice (n = 16) were more immobile (s) (**a**), made fewer turn angles (degrees) (**b**), made fewer rotations (n) (**c**) and had reduced mobility (s) (**d**) and a larger fraction of the α_2_^+/G301R^ mice emitted audible squeaks (given as vocalization (%)) during the experiment (**e**) compared to WT mice (n = 21). (**f,g**) Stress-induced sucrose preference test of α_2_^+/G301R^ mice (n = 15) and WT mice (n = 13) showed that two stress protocols (Stress I and II) had a higher influence on the relative sucrose intake (%) of α_2_^+/G301R^ mice than that of WT mice. Cages with reduced relative sucrose intake (%) compared to unstressed (none) were denoted anhedonic, a, and marked with red filling (**f**). The liquid intake given as liquid/mouse/day (**g**) was comparable for cages harboring α_2_^+/G301R^ and WT mice (**g**). Data shown as means ± s.e.m. ★*P* < 0.05, ★★*P* < 0.01, ★★★*P* < 0.001.

The tail suspension test (TST), showed that α_2_^+/G301R^ mice exhibited a stress-induced depression-like phenotype as they displayed increased immobility, reduced mobility, reduced movement, and fewer rotations compared to WT mice (Fig. 6a–d). Furthermore, mice become vocal in stressful situations^56^, and during the TST ~50% of α_2_^+/G301R^ mice emitted audible squeaks compared to ~14% of WT mice (Fig. 6e).

The sucrose-preference test models anhedonia^44^, and unstressed α_2_^+/G301R^ and WT mice had a similar and high (~98%) preference for sucrose-solution over water (“none”, Fig. 6f). The α_2_^+/G301R^ mice displayed anhedonia when short- and long-lasting stress protocols were applied prior to the test. Firstly, after a short-lasting stress protocol, ~67% of cages housing α_2_^+/G301R^ mice reduced their relative sucrose intake compared to 0% of cages housing WT mice (stress I, Fig. 6f). Next, a long-lasting stress-protocol resulted in reduced relative sucrose intake in ~83% of cages housing α_2_^+/G301R^ mice compared to ~20% of cages housing WT mice (stress II, Fig. 6f). The relative sucrose intake was rarely below ~90% in any cages, and this can be ascribed to the lack of social stress (no individual housing). The overall liquid intake was largely the same in all mice cages during the experiment (Fig. 6g).

In summary, the FHM2 mouse model harboring the G301R mutation mimics FHM2-relevant psychiatric manifestations, and further associate the α_2_Na^+^/K^+^-ATPase to both glutamate and female sex hormone systems (Table 1).

## Discussion

FHM is an autosomal dominantly inherited subtype of migraine with aura, characterized by transient neurological signs and symptoms. FHM2 is a rare disease and, although more severe, it manifests largely as MA and fulfils the classic migraine criteria^57^. A genome-wide linkage scan showed a susceptibility locus for common migraine on chromosome 1q, and *ATP1A2* (or a flanking gene) was hypothesized to be involved in common migraine besides FHM2^58^. Common migraine was recognized as the seventh disabler in the Global Burden of Disease Survey 2010 affecting ~14% of adults, and is the most costly neurological disorder^59^. The episodic nature of migraine attacks makes the disease difficult to treat and prevent. Moreover, in the majority of cases, migraine is caused by a poorly understood interplay of both genetic and environmental factors, and the frequent co-occurrence of a variety of other manifestations further complicates the pathological picture.

To reach a better understanding of the pathological spectrum of migraine, knowledge of the molecular pathway(s) involved and the co-morbid manifestations are warranted. To address these issues, we generated a knock-in mouse model introducing the FHM2-causing G→A mutation in the *Atp1a2* gene encoding the FHM2 mutation, α_2_G301R. The analyses of α_2_-protein expression all suggest that FHM2 is caused by α_2_ haploinsufficiency, with WT protein levels of the brain-expressed paralogs α_1_ and α_3_ isoforms in brain stem (BS), hippocampus (HS), cerebellum (CRBL) and cortex (CTX). These results are comparable to the expression of α_1_ and α_3_ isoforms in total brain lysates reported for the α_2_ W887R knock-in mouse model^19^.

Our results showed that the α_2_^+/G301R^ mouse displayed several behavioral phenotypes, which have relevance for FHM2/MA, co-morbid mood depression and OCD. Moreover, these manifestations occurred in patients from two FHM2-families harboring the α_2_G301R mutation^4^,^20^. Both male and female α_2_^+/G301R^ mice showed specific behavioral phenotypes most likely coupled to imbalance in the glutamate system and probably involving the NMDA receptor, and a mechanism(s) behind FHM2 may be the result of reduced glutamate clearance by astrocytes involving the α_2_Na^+^/K^+^-ATPase^11^,^12^. We provide evidence that both genetic (the *Atp1a2* G301R mutation) and physiological (female sex hormone) factors modulate co-morbid psychiatric manifestations relevant to FHM2. Both clinical and epidemiological data on hormonal modulation of FHM2-related manifestations are limited, but other lines of evidence towards hormonal influence of FHM-related phenotypes exist, and are described below in relation to observed phenotypes for the α_2_^+/G301R^ mice.

The initiation of CSD is largely dependent on high levels of extracellular glutamate and K^+^ ions^15^. The K^+^ ions are released after neuronal activity, and believed to accumulate due to decreased removal of K^+^ and glutamate by astrocytes. This leads to a wide-arching depolarization which compromise further neuronal firing. The NMDA receptor is a prime “target” for excessive extracellular glutamate, and this receptor does play an important role in the initiation, propagation and duration of CSD^60^. Although the male α_2_^+/G301R^ mice showed CSD propagation speed and cerebral blood flow measurements comparable to male WT mice, this is not conclusive as several factors effect the variation of the propaganda speed (e.g, a CSD wave can spread in several directions from the point of induction and not necessarily move in a straight linear direction, which means that the time it takes for the CSD to spread from the site of elicitation can differ between the two electrodes. Moreover, the alignment of the two electrodes may be affected by e.g presence of blood vessels). However, the electrocorticography revealed that the recovery phase after CSD was affected.

In comparison, the α_2_^+/W887R^ KI mouse model displayed a decreased threshold for CSD induction and an increased CSD velocity of propagation^19^. The differences between these studies, and the studies presented here, likely reflects the different assay conditions i.e., KAc was used for CSD induction in the right somatosensory cortex in the male α_2_^+/G301R^ mice, whereas incremental current stimuli were delivered up to CSD induction in the occipital cortex in the α_2_^+/W887R^ KI mice. Moreover, the different use of anesthetics (α-chloralose versus urethane) could also explain the differences observed. Notably, previous studies of other anesthetics were shown to influence the CSD induction threshold and propagation speed differently^61^.

Moreover, in line with a previous study of two FHM1 knock-in mouse models^49^, the level of recovery of evoked potentials 15 min. after CSD is the same in WT and α_2_^+/G301R^ mice, indicating that the spontaneous activity (ECoG signal) is affected whereas the evoked activity is not. The fact that the evoked potentials do not fully recover to *pre*-CSD levels, as observed for the two FHM1 knock-in mouse models^49^, probably reflects that some of the mechanisms that underlie this unfiltered EEG signal have not recovered within the time frame of the experiment. Further study of two different FHM1 knock-in mouse models showed that the female knock-in mice were more susceptible to CSD^49^ than male knock-in mice, and this sex difference was abolished by ovariectomy and senescence, proving a role for the female sex hormone cycle—besides the genetics—in the susceptibility to CSD. In this context, it is interesting that the α_2_^+/G301R^ mice displayed additional female-specific behaviors related to both the glutamate system and the female sex hormone cycle, indicating that female α_2_^+/G301R^ mice might be more sensitive to the (molecular) alterations that the α_2_G301R-mutation cause, and thus, the penetrance of specific behavioral phenotypes might therefore be higher compared to male α_2_^+/G301R^ mice.

There is a preponderance of females among migraineurs suffering from FHM, sporadic hemiplegic migraine (SHM) and familial non-hemiplegic MA^5^,^62^. The levels of total estrogen and estradiol were elevated in migraineurs with aura at the two stages (menses and ovulation) investigated compared to control group^63^. Notably, MA has been considered an absolute contraindication to the use of combined hormonal contraception^64^, and preliminary results showed that progestin-only contraception had a positive effect in the course of MA, reducing the number of days with migraine^65^. Several of the female-specific behaviors of α_2_^+/G301R^ mice were rescued to WT behaviors by a single Depoprovera treatment that reduced/stabilized the estrogen levels. A previous study has shown that treatment of fertile rats with a raloxifene-analog reproduced the effects observed after ovariectomy, proving that the raloxifene-analog functioned as an anti-estrogen in the cerebral brain areas investigated^66^. Moreover, *in vitro* studies of raloxifene-treated astrocytes demonstrated an increase in glutamate uptake^14^, showing that an anti-estrogen effect mediated by the estrogen receptor enhances glutamate clearance of astrocytes.

There is increasing evidence that alterations in glutamatergic neurotransmission contributes to migraine^67^. EAAT2 is predominantly expressed in astrocytes and is crucial for glutamate uptake in the central nervous system^68^. Moreover, previous *in vitro* studies indicated a role for the α_2_Na^+^/K^+^-ATPase, in complex with EAAT1 and EAAT2, in astroglial glutamate uptake, i.e., glutamate clearance from the synaptic cleft^11^,^12^. We showed that glutamate uptake is impaired *in vitro* in hippocampal-derived matured mixed cultures of astrocytes and neurons obtained from α_2_^G301R/G301R^ E17 embryonic mice compared to WT. In mixed cultures, neurons will support glutamate uptake, but to a much lower degree than the astrocytes^69^. Although embryonic and adult neurons do express the α_3_ isoform (which has a lower sodium affinity than the α_2_ isoform), and thus, could support sodium-coupled glutamate uptake, it is unlikely that the difference in glutamate uptake observed between the mixed cultures derived from α_2_^G301R/G301R^ homozygous and WT E17 embryonic mice, represents a difference in neuronal glutamate uptake.

The α_2_ isoform is expressed in a differential developmental manner: the α_2_ isoform is mainly found in astrocytes and in a small subset of hippocampal neurons in the adult brain^7^, which is in contrast to the newborn brain, where the α_2_ isoform is predominantly found in neurons^70^. This indicates that the reduced glutamate uptake in the matured mixed astrocyte and neurons cultures from the α_2_^G301R/G301R^ mice (mimicking adult conditions) should mostly be attributed to the lack of a functional α_2_Na^+^/K^+^-ATPase in astrocytes, however, lack of a functional α_2_Na^+^/K^+^-ATPase in the subset of neurons, which would normally express a functional α_2_Na^+^/K^+^-ATPase, could also account for a fraction of the reduced glutamate uptake observed.

Moreover, we observed higher levels of glutamate in lysates from various brain areas from adult female α_2_^+/G301R^ mice compared to lysates from WT mice, although, it remains to be elucidated whether the increased glutamate level is derived from extra- and/or intracellular compartments. Altogether, these data support that the molecular mechanism behind FHM2 is coupled to the glutamate system and coupled to reduced glutamate clearance of astrocytes involving the α_2_Na^+^/K^+^-ATPase. Estradiol has previously been shown *in vitro* to enhance depolarization-induced presynaptic glutamate release^71^ and *in vivo* to increase both the total glutamate level and the extracellular glutamate level in the arcuate nucleus^72^. Our results are in agreement with the hypothesis that estrogen plays a significant role in the auto-regulation of brain (and blood) glutamate levels^73^. Furthermore, our results suggest that high levels (and/or fluctuating levels) of estradiol might aggravate the effects on the glutamate system caused by haploinsufficiency of the gene encoding the α_2_-isoform of the Na^+^/K^+^-ATPase.

Studies of migraineurs with aura (between attacks) and migraine-prone children showed that they exhibited an increased responsiveness to aversive acoustic stimuli^74^,^75^. Increased ASR behavior is also observed in patients with OCD^76^, and, to date, adult females from two different FHM2-families—one harboring the α_2_G301R mutation—have been diagnosed with OCD besides FHM2^4^,^77^. Moreover, accumulating evidence (neuroimaging, animal models, candidate gene and treatment studies) points to the fact that glutamate system defects might lead to OCD^78^,^79^, which is in agreement with cerebrospinal fluid from OCD-affected adults containing increased glutamate levels compared to controls^80^. Notably, different psychiatric manifestations (including OCD) are reported to be co-morbid to MA^81^.

A human study based on questionnaires to female outpatients with primary OCD showed that reproductive cycle events do influence the symptom severity of OCD^82^. The female-specific α_2_^+/G301R^ mice behaviors mimicking compulsive behaviours of OCD could be rescued to WT behaviors by Depoprovera and memantine (an NMDA receptor antagonist^83^), whereas OCD-related female- and male-specific α_2_^+/G301R^ mice behavior could be rescued to WT behavior by treatment with amantadine (an NMDA receptor antagonist^84^). Our results showed that behaviors likely resulting from lack of proper glutamate clearance of astrocytes could be circumvented by treatment with amantadine or memantine, which both are proposed to allow normal synaptic transmission but decreasing over-activation of NMDA-receptors^84^,^85^. Selective serotonin reuptake inhibitors (SSRIs) are currently the first-line OCD-treatment, however, ~40–60% of OCD-patients remain refractory to treatment with SSRIs^86^.

The association between anxiety and migraine has been observed in both clinic- and community-based studies^87^. Moreover, an epidemiological study reported the presence of anxiety disorders in 75.8% of the OCD patients studied^88^. Notably, mild anxiety was reported in an FHM2-family during attacks^89^, however, the α_2_^+/G301R^ mice did not appear anxious in the EPM assay. This is different compared to previous FHM2 mouse models, which showed an increased susceptibility to fear and anxiety^19^,^23^,^90^ and most likely, this could be either attributed to different mouse inbred strains, or the fact that different exons and mutations were knocked-out or introduced, respectively, mimicking the complex genotype-phenotype in FHM2 patients. To date, social phobia has not been reported for any FHM2 family. However, a population study showed association between migraine and social phobia^91^. Moreover, an epidemiological study reported a 43.5% prevalence of social phobia/anxiety in OCD making this anxiety disorder the most frequently observed anxiety disorder co-morbid to OCD^88^. The α_2_^+/G301R^ mice showed behavior characteristic for decreased sociability, supporting clinical findings for migraine and OCD patients.

Population studies demonstrated that depression was co-morbid with migraine^92^, and mood depression was described for a FHM2-family harboring the α_2_G301R-mutation^4^. Moreover, an epidemiological study reported that 63.3% of OCD-patients suffered from a mood disorder besides OCD^88^. In line with these studies, the α_2_^+/G301R^ mice exhibited stress-induced depression. Altogether, our data support the opinions that co-morbid psychiatric manifestations to migraine/FHM2 should be considered part of the pathological spectrum.

The behavioral phenotypes of α_2_^+/G301R^ mice are likely caused by an intricate interplay of effects that are generated by reduced glutamate clearance from the synaptic cleft, and they seem to be aggravated and extended in female α_2_^+/G301R^ mice by effects of the female sex hormone cycle hereon. The alterations in the glutamate system in male and female α_2_^+/G301R^ mice and the role of the female sex hormone cycle in female-specific α_2_^+/G301R^ mice behaviors, open up a range of possibilities for developing optimal strategies to successfully treat FHM2/migraine patients with co-morbid psychiatric manifestations.

Some clinical limitations apply to our study. Firstly, the experiments were performed in mice, which are imperfect models of human neurological disorders. Secondly, a few studies exist describing the therapeutic effects of different NMDA receptor antagonists on migraine or migraine related symptoms^93^,^94^, however, it is uncertain if FHM2 patients would benefit from treatment with amantadine, memantine or other NMDA receptor antagonists. Interestingly, a recent study showed that amantadine could improve obsessive compulsive symptoms when used as an adjunctive therapy to selective serotonin reuptake inhibitors (SSRIs) in OCD-patients refractory to SSRI pharmacotherapy alone^95^. These results support the hypothesis that dysfunctional transduction of the glutamate signal via the NMDA receptor play a role in OCD, and thus, may play a similar role in OCD co-morbid to FHM2. Thirdly, the female sex hormone cycle might prove valuable as a target for treatment of females suffering from FHM2 and/or common migraine, however, this will require further clinical testing.

## Additional Information

**Data availability**: The α2+/G301R mouse model is available through a Material Transfer Agreement (MTA).

**How to cite this article**: Bøttger, P. *et al.* Glutamate-system defects behind psychiatric manifestations in a familial hemiplegic migraine type 2 disease-mutation mouse model. *Sci. Rep.* **6**, 22047; doi: 10.1038/srep22047 (2016).

## Supplementary Material

Supplementary Information

## Figures and Tables

**Table 1:** Summary of major phenotypes and pharmacological treatments.

Phenotype/Treatment	α_2_^+/G301R^
CSD	Prolonged recovery phase after CSD induction (males)
Glutamate system	Elevated glutamate levels in adult brain regions (females)
	Reduced [^3^H]-D-aspartate (mixed cultures of neurons and astrocytes from α_2_^G301R/G301R^ (females and males)
OCD	Increased ASR (females and males)
	Increased grooming behaviour (females)
	Increased marble-burying behavior (females)
Depression	Stress-induced depression-like phenotype (females and males)
	Anhedonia (females and males)
Sociability	Reduced sociability (females and males)
Pharmacological treatments	*Depoprovera (suppresses the female sex hormone cycle)* Rescued hypoactivity (females) Rescued grooming (females) Rescued marble burying behavior (females)
	*Amantadine (glutamate antagonist)* Rescued ASR (females and males) Rescued marble burying behavior (females)
	*Memantine (glutamate antagonist)* Rescued marble burying behavior (females)
