# Supplementary material for: Glutamate-system defects behind psychiatric manifestations in a familial hemiplegic migraine type 2 disease-mutation mouse model
Source: Sci Rep. 2016 Feb 25;6:22047. doi: 10.1038/srep22047 (PMC4766516; doi:10.1038/srep22047)
Supplement: Supplementary Information [file srep22047-s1.pdf]

Glutamate-system defects behind psychiatric manifestations in a familial hemiplegic migraine type 2 disease-mutation mouse model

Pernille Bøttger[1,2,3], Simon Glerup[1,4], Bodil Gesslein[5], Nina B. Illarionova[6], Toke J. Isaksen[1,2], Anders Heuck[1,2], Bettina H. Clausen[3], Ernst-Martin Füchtbauer[7], Jan B. Gramsbergen[3], Eli Gunnarson[6], Anita Aperia[6], Martin Lauritzen[5,8], Kate L. Lambertsen[3], Poul Nissen[2,7,9], Karin Lykke-Hartmann[1,2,10*]

Affiliations:
[1]Aarhus University, Department of Biomedicine, DK-8000 Aarhus, Denmark.
[2]Centre for Membrane Pumps in Cells and Disease-PUMPKIN, Danish National Research Foundation, Aarhus University, Department of Molecular Biology and Genetics, DK-8000 Aarhus C, Denmark.
[3]University of Southern Denmark, Institute of Molecular Medicine, Department of Neurobiology Research, DK-5000 Odense, Denmark.
[4]The Lundbeck Foundation Research Centre MIND, Aarhus University, Department of Biomedicine, DK-8000 Aarhus C, Denmark.
[5]University of Copenhagen, Department of Neuroscience and Pharmacology and Center for Healthy Aging, DK-2200 Copenhagen N, Denmark.
[6]Karolinska Institutet, Department of Women's and Children's Health, SE-171 76 Stockholm, Sweden.
[7]Aarhus University, Department of Molecular Biology and Genetics, DK-8000 Aarhus, Denmark.
[8]Glostrup Hospital, Department of Clinical Neurophysiology, DK-2600 Glostrup, Denmark.
[9]Danish Research Institute for Translational Neuroscience-DANDRITE, Nordic-EMBL Partnership of Molecular Medicine, Aarhus University, Department of Molecular Biology and Genetics and Department of Biomedicine, DK-8000 Aarhus C, Denmark.
[10]Aarhus Institute of Advanced Studies, Aarhus University, Høegh-Guldbergs Gade 6B DK-8000 Aarhus C, Denmark

* Correspondence: Dr K Lykke-Hartmann, Aarhus Institute of Advanced Studies and Department of Biomedicine Aarhus University, Wilhelm Meyers Allé 4, DK-8000 Aarhus C, Denmark, Tel: +45-29390558, Fax: +45-86128804, E-mail: kly@biomed.au.dk.

Supplementary Table 1. Generation and basic characterization of the *Atp1a2* G301R knock-in mouse

Fig. S1a

Aim	Cloning information	Procedure information
Vector background	StrataCloneTMpSC-B 3.5kb (Stratagene Corp) using StratagClone Blunt CPR (#200184)	
pSC-B-N (intron 6 to intron 8)	P1: 5´- CGG ATC GAT GCA GCC CAG AGA TGA AAG AC -3´<br>P2: 5´- CGG GCG GCC GCT GAC ACA TCA CAG TGG CTG A -3´<br><br>3.38 kb fragment from 129Svj DNA	PfuUltra fusion HS DNA polymerase (Stratagene Corp, #600670-12)
pSC-B-C (intron 7 to intron 13)	P3: 5´- CCA ACT GTT AGG AGT ATG GAA GTC AAG TGA G -3´<br>P4: 5´- GCT CTA ATA AAC CTT TAG TTT ATA CTC ATG TGC TAA TG -3´<br><br>6.68 kb fragmet from 129Svj DNA	
pSC-B-N LoxP-Neo-LoxP (*Afl*II)	LoxP-Neo-LoxP (1.5kb) *Cla*I-digested from pLoxP-PGK-Neo vector (4.512kb)	Cloned blunt in into the *Afl*II-digested pSC-B-N vector (intron 7)
pSC-B-N-loxP-Neo-LoxP (*Afl*II)- LoxP (*Bst*EII)	pSC-B-N PCR fragment (564bp):<br><br>P5: 5´- ATT AAC CCT CAC TAA AGG GA -3´<br>P6: 5´- CAT CTC TAG AAT TGT TGG CCA CCG TGA CTG T -3´<br>P7: 5´- GTC ACA TAA CTT CGT ATA ATG TAT GCT ATA CGA AGT TAT -3´<br>P8: 5´- GTG ACA TAA CTT CGT ATA GCA TAC ATT ATA CGA AGT TAT -3´	The 564bp PCR fragment was subcloned. Annealed LoxP oligos (P7 and P8) cloned into this, using *Bst*EII restriction sites. This partial pSC-B-partial-N-LoxP (*Bst*EII) was digested with *Hind*III and *Msc*I (518bp) and the 518 bp fragment was cloned into the *Hind*II- and *Msc*I-digested pSC-B-N LoxP-Neo-LoxP (*Afl*II), generating pSC-B-N-LoxP-Neo-LoxP (*Afl*II)-LoxP(*Bst*EII).<br>*Hind*III(R0104S) and *Stu*I (R0187S) from New England Biolabs.
Mutagenesis of G_>A encoding the G301R mutation (exon 8)	PCR-1 generates fragment of 623 bp<br>P9: 5'- CGA TAA GCT TGA TAT CCA CTG TGG AA -3' (*Hind*III)<br>P10: 5'- CTG TGT TCC TG**A** GGG TCT CCT TCT -3'<br>PCR-2 generates fragment of 1 097 bp<br>P11: 5´- AGA AGG AGA CCC **T**CA GGA ACA CAG -3´<br>P12: 5´- CCA GGC CTA CCC GCT TCC ATT GCT C -3´ (*Stu*I)<br>PCR-3: template is fragments from PCR´s 1 and 2<br>P9: 5'- CGA TAA GCT TGA TAT CCA CTG TGG AA -3'<br>P12: 5´- CCA GGC CTA CCC GCT TCC ATT GCT C -3´	Pfu DNA polymerase (EP0572, Fermentas^{TM}) generated PCR 3, was *Hind*III and *Stu*I-digested and the 622 bp product was subcloned into the *Hind*III and *Stu*I-digested pSC-B-"N"-"LoxP-NEO-LoxP(*Afl*II)"-"LoxP(*Bst*EII)", generating the pSC-B-"N"-"LoxP-NEO-LoxP(*Afl*II)"-"LoxP(*Bst*EII)"-"G→A" vector.
Combination of "N" and "C"	Removal of pSC-B vector sequence flanking *Hind*III-site in pSC-B-"N"-"LoxP-NEO- LoxP(*Afl*II)"-"LoxP(*Bst*EII)"-"G→A".<br>P13: 5´- GCA AGC TTA GCT GAG AAA TGT CAA TGG ATT TG -3´ (*Hind*III) P14: 5´- CGG TGG ATG TGG AAT GTG TG -3´<br>PCR generates fragment of 1 406 bp which was *Hind*III and *Bst*EII digested and a fragment of 622 bp was purified	*Hind*III and *Bst*EII-digested pSC-B-"N"-"LoxP-NEO-LoxP(*Afl*II)"-"LoxP(*Bst*EII)"-"G→A" was SAP-treated and fragment purified after agarose gel separation. The 622 bp fragment was sticky- end ligated into pSC-B-"N"-"LoxP-NEO-LoxP(*Afl*II)"-"LoxP(*Bst*EII)"-"G→A" (SAP, *Hind*III and ligase informations above). This generates pSC-B-"N"-"LoxP-NEO-LoxP(*Afl*II)"-"LoxP(*Bst*EII)"-"G→A"-"trim"
	PCR generates a 4.39 kb fragment of pSC-B-"C"<br>P15: 5´- CTC ATC CTT AAT GCT GCT GC-3´<br>P16: 5´- TGC TCT AAT AAA CCT TTA GTT TA-3´	*Hind*III-digestion of pSC-B-"N"-"LoxP-NEO- LoxP(*Afl*II)"-"LoxP(*Bst*EII)"-"G→A"-"trim" generated a 4.39 kb fragment -> blunt-end ligated into pSC-B-"N"-"LoxP-NEO-LoxP(*Afl*II)"-"LoxP(*Bst*EII)"-"G→A"-"trim". This generates pSC-B-"N"-"LoxP-NEO-LoxP(*Afl*II)"-"LoxP(*Bst*EII)"-"G→A"-"trim"-"C"
2×TK	2×TK amplified from plasmid (pBSSK-TK2-14) by PCR generating *Sac*II sites. P17: 5´- CGC ACC GCG GGT AAT ACG ACT CAC TAT AGG -3´ (*Sac*II)<br>P18: 5´- GCG TGG CGC CAA TTA ACC CTC ACT AAA GGG AA -3´ (*Sac*II)<br><br>PCR product of 3652 bp is digested with *Sac*II and purified	Plasmid pBSSK-TK2-14 from DAGMAR Facility, AU.<br>*Sac*II-digested pSC-B-"N"-"LoxP-NEO-LoxP(*Afl*II)"-"LoxP(*Bst*EII)"-"G→A"-"trim"-"C" was purified. The 2×TK fragment was ligated into pSC-B-"N"-"LoxP-NEO-LoxP(*Afl*II)"-"LoxP(*Bst*EII)"-"G→A"-"trim"-"C". This generates the final targeting vector of 16.69 kb.

Fig. S1b

Test	Aim	Primers	Genotype	Target	Genomic DNA source	PCR: (lengths, bp)	Information
PCR	Homologous recombination	P19: 5´- ATC AGC GAG CTC AGG ACA TT -3´ P20: 5´- CAG GAC GTG ACA AAT GGA AG -3´<br>P19 locates to exon 4 outside targeting construct<br>P20 locates to intron 7 (NEO-specific)	WT (+/+)	5´end: Exon 4 to intron 7	C1: 129/SvJ	0	Template: 200 ng<br>DMSO: 1%<br>Long PCR enzyme mix
			WT (+/+)		C2: MDX/BL6	0	
			KI (+/-)		IIH6 ES cell clone	4 679	
		P21: 5´- TGC TAT TGT CTT CCC AAT CCT -3´ P22: 5´- GTG GCA GAA TGG AGC AAG TC -3´<br>P21 locates to intron 7 (NEO-specific)<br>P22 locates to intron 13 outside targeting construct	WT (+/+)	3´end: Intron 7 to intron 13	C1: 129/SvJ	0	Template:50 ng<br>DMSO: 4%<br>Long PCR enzyme mix
			WT (+/+)		C2: MDX/BL6	0	
			KI (+/-)		IIH6 ES cell clone	7 595	
		P20: 5´- CAG GAC GTG ACA AAT GGA AG -3´ P21: 5´- TGC TAT TGT CTT CCC AAT CCT -3´<br>P20 locates to intron 7 (NEO-specific) P21 locates to intron 7 (NEO-specific)	WT (+/+)	NEO: Intron 7	C1: 129/SvJ	0	Template: 200 ng<br>Long PCR enzyme mix (K0182, Fermentas^{TM})
			WT (+/+)		C2: MDX/BL6	0	
			KI (+/-)		IIH6 ES cell clone	1 329	

Supplementary Table 1. Generation and basic characterization of the *Atp1a2* G301R knock-in mouse – *continued*

Test	Aim	Primers (and probe-related information)	Genotype	Genomic DNA source	Reagents	Genomic DNA: *Kpn*I and *Spe*I digest (probed lengths, bp)	Information	Figure
Southern blot	Homologous recombination (5´ end)	Probe length: 500 bp (template: plasmid with 129/SvJ genomic DNA)<br>P19: 5´- ATC AGC GAG CTC AGG ACA TT - 3´ P23: 5´- CAG TCT CCT AAC CAC GCT CC -3´	WT (+/+)	ES cells	*Kpn*I enzyme (R0142S) *Spe*I enzyme (R0133S) New England Biolabs Ltd	9 692	Double-digest of approx. 5-10 µg genomic DNA using *Kpn*I and *Spe*I	S1c
			KI (+/-)	IIH6 ES cells		9 692; 11 340		

Test	Aim	Primers (and PCR-related information)	Genotype	Genomic DNA source	PCR: (length, bp) *Bst*EII-digest	Information	PCR: (length, bp) *Bst*EII and *Bgl*II-digest	Figure
PCR	Partial Cre-enzyme treatment	P3: 5´- CCA ACT GTT AGG AGT ATG GAA GTC AAG TGA - 3´ P24: 5´- GAG GGA TAC AGT ATG GGA AC -3´<br>P3 locates to intron 7<br>P24 locates to intron 11<br>Template: 50 ng<br>Long PCR enzyme mix<br>(K0182, Fermentas^{TM}) Thermo Fisher Scientific Inc	KI (+/-)	IIH6Cre14 (Partial Cre)	1 632/1 610/1 599; 1 031; 546/506 ~ 3 bands	*Bst*EII enzyme (R0162S) *Bgl*II enzyme (R0144S) New England Biolabs Ltd	1 610; 1 032; 935; 902; 697; 546/506 ~ 6 bands	S1d
			KI (+/-)	C3 (Full Cre)	2 021; 1 599; 1 031; 506/546 ~ 4 bands		1 324; 1 031; 902; 697; 546/506 ~ 5 bands	
			WT	C4: 129/SvJ (WT)	1 599; 1 031; 546/506 ~ 3 bands		1 031; 902; 697; 546/506 ~ 4 bands	

Test	Aim	Primers	Genotype	PCR: (length, bp)	Reagents	PCR: *Bst*EII-digest (length, bp)	Information	Figure
PCR	Genotyping	P6: 5'- CAT CTC TAG AAT TGT TGG CCA CCG TGA CTG T -3' P24: 5'- GAG GGA TAC AGT ATG GGA AC -3'<br>P6 locates to exon 8/intron 8<br>P24 locates to intron 11	WT (+/+)	1 876	DreamTaq DNA polymerase (EP0702, Fermentas^{TM}) Thermo Fisher Scientific Inc<br>*Bst*EII enzyme (R0162S) New England Biolabs Ltd	1 031; 516; 329	Abolishment of *Bst*EII-site due to 3´ LoxP (33 bp) insertion in intron 8	S1e
			KI (+/-)	1 876/1 909		1 393; 1 031; 516; 329		
			KI (-/-)	1 909		1 393; 516		

Test	Aim	Primer	Genotype	Amount of genomic DNA (ng/μL)	Information	Figure
Sequencing	Detection of G->A mutation	P25: 5´- CAG TCA TTG GCA AAC CCA G -3´ P25 locates to intron 7	KI (+/-)	10 ng/µL (15 µL)	Sequencing (Eurofins MWG Operon facility, Germany)	S1f
			WT (+/+)	10 ng/µL (15 µL)		

Test	Sex (F/M)	Measurement	Genotype/ Animals (n)	Mean and s.e.m.	Statistical test	*P*-value	Figure
Body weight	F	Body weight (g)	WT = 7	Not shown	Two-way ANOVA	Genotype: *P* = 0.928	S1g
			KI = 11	Not shown			
	M		WT = 7	Not shown	Two-way ANOVA	Genotype: *P* = 0.004	
			KI = 6	Not shown			

Supplementary Table 2. Western blotting

Test	Age (days)	Sex (F/M)	Genotype	Load (µg pr lane)	α_2-protein amount (Arbitrary Units, AU)	GAPDH-protein amount (Arbitrary Units, AU)	Reagents (or indicated information)	Relative α_2-level quantification (%)	Information	Figure
SDS-page Western blot	E17.5	-	WT (+/+)	30	3 355 752	2 732 798	Rabbit anti human α_2 aa 432-455 (07674, Upstate) Millipore Corp 1:5 000 dilution Rabbit anti human GAPDH aa 1-335 (sc-25778) Santa Cruz Biotechnology	100	α_2-isoform:~ 110 kDa GAPDH:~ 35 kDa	S2a
			KI (+/-)	30	2 299 512	2 422 280		77		
			KI (-/-)	30	962 072	2 724 203		29		
	New born (0 days)	-	WT (+/+)	30	11 839 549	67 277 802		100		
			KI (+/-)	30	6 119 085	59 089 772		59		
			KI (-/-)	30	431 997	56 240 436		4		
	200	M	WT (+/+)	25	192 587	9 643 299	Hippocampus, HC	100	Brain areas were dissected from two mice of each genotype and pooled	
			KI (+/-)	25	123 548	9 724 048		64		
			WT (+/+)	25	257 109	8 228 897	Cerebellum, CRBL	100		
			KI (+/-)	25	118 848	9 506 595		40		
			WT (+/+)	25	155 539	9 766 206	Cortex, CTX	100		
			KI (+/-)	25	107 716	11 050 225		61		
			WT (+/+)	25	260 909	6 267 243	Brain stem, BS	100		
			KI (+/-)	25	90 246	9 842 882		22		
	Adult	M+F	C5: WT brain lysate	25	-	-	Whole brain	-	Pool of two mice brains	
SDS-page Western blot	E17.5	-	WT (+/+) = 1	30	3 355 752 (AU)	2 732 798 (AU)	Rabbit anti human α_2 aa 432-455 (07674, Upstate) Millipore Corp 1:5 000 dilution Rabbit anti human GAPDH aa 1-335 (sc-25778) Santa Cruz Biotechnology Inc 1:200 dilution	100	α_2-isoform:~ 110 kDa GAPDH:~ 35 kDa	S2a
			KI (+/-) = 1	30	2 299 512 (AU)	2 422 280 (AU)		77		
			KI (-/-) = 1	30	962 072 (AU)	2 724 203 (AU)		29		
		-	WT (+/+) = 1	30	127 484 (QL)	15 267 001 (QL)		91		-
			WT (+/+) = 1	30	117 625 (QL)	11 813 043 (QL)		109		
			KI (+/-) = 1	30	108 806 (QL)	19 488 227 (QL)		56		
			KI (+/-) = 1	30	54 176 (QL)	16 568 309 (QL)		33		
			KI (-/-) = 1	30	44 384 (QL)	25 354 080 (QL)		18		
			KI (-/-) = 1	30	26 650 (QL)	20 449 931 (QL)		13		
	E17.5	-	WT (+/+) = 3	See above	See above	See above	See above	100 ±5	See above	S2c
			KI (+/-) = 3					55 ±13		
			KI (-/-) = 3					20 ±5		
	New born (0 days)	-	WT (+/+) = 1	30	11 839 549 (AU)	67 277 802 (AU)	Rabbit anti human α_2 aa 432-455 (07674, Upstate) Millipore Corp 1:5 000 dilution Rabbit anti human GAPDH aa 1-335 (sc-25778) Santa Cruz Biotechnology Inc 1:200 dilution	100	α_2-isoform:~ 110 kDa GAPDH:~ 35 kDa	S2b
			KI (+/-) = 1	30	6 119 085 (AU)	59 089 772 (AU)		59		
			KI (-/-) = 1	30	431 997 (AU)	56 240 436 (AU)		4		
		-	WT (+/+) = 1	30	38 583 437 (AU)	79 240 591 (AU)		100		-
			KI (+/-) = 1	30	13 640 643 (AU)	46 778 927 (AU)		60		
		-	WT (+/+) = 1	30	95 802 (QL)	134 502 (QL)		109		
			WT (+/+) = 1	30	93 790 (QL)	168 938 (QL)		85		
			WT (+/+) = 1	30	153 979 (QL)	221 425 (QL)		106		
			KI (+/-) = 1	30	78 500 (QL)	205 570 (QL)		58		
			KI (+/-) = 1	30	82 460 (QL)	195 920 (QL)		64		
			Ki (+/-) = 1	30	75 448 (QL)	186 024 (QL)		62		
			KI (-/-) = 1	30	13 104 (QL)	197 134 (QL)		10		
	New born (0 days)	-	WT (+/+) = 5	See above	See above	See above	See above	100 ±4	See above	S2c
			WT (+/-) = 5					61 ±1		
			WT (-/-) = 2					7 ±3		

Supplementary Table 2. Western blotting – *continued*

Test	Age (days)	Sex (F/M)	Genotype	Load (µg pr lane)	α_2-protein amount (Arbitrary Units, AU)	GAPDH-protein amount (Arbitrary Units, AU)	Reagents (or indicated information)	Relative α_2-level quantification (%)	Information	Figure
SDS-page Western blot	New born (0 days)	-	WT (+/+)	30	2 024 365	2 932 729	See above for antibodies	100	See above for expected protein sizes	S2b
			KI (+/-)	30	1 205 054	2 867 534		61		
			KI (-/-)	30	208788	3 330 428		9		
	188-197	F	WT (+/+)	25	68558	2 017 685	Hippocampus, HC	100	Brain areas were dissected from two mice of each genotype and pooled	
			KI (+/-)	25	48008	2 248 450		63		
			WT (+/+)	25	97 58	1 620 436	Cerebellum, CRBL	100		
			KI (+/-)	25	73674	1 807 271		68		
			WT (+/+)	25	75862	2 172 274	Cortex, CTX	100		
			KI (+/-)	25	68164	2 450 003		80		
			WT (+/+)	25	173217	1 575 130	Brain stem, BS	100		
			KI (+/-)	25	79042	1 614 753		45		
	Adult	M+F	C5: WT brain lysate	25	-	-	Whole brain	-	Pool of two mice brains	
SDS-page Western blot	Adult	F	WT (+/+)	20	148208	155029	Brain stem, BS (pool from two mice)	100	Mouse anti chicken α_1 polypeptide of 1020 aa (DM Fambrough, DSHB) 1:1 000 dilution<br><br>Rabbit anti human GAPDH aa 1-335 (sc-25778) Santa Cruz Biotechnology Inc 1:200 dilution<br><br>Molecular weights: α_1-isoform: ~ 110 kDa GAPDH:~ 35 kDa	-
			KI (+/-)	20	149972	151417		104		
			WT (+/+)	20	133690	162681	Hippocampus, HC (pool from two mice)	100		
			KI (+/-)	20	121805	134875		110		
			WT (+/+)	20	212654	153383	Cerebellum, CRBL (pool from two mice)	100		
			KI (+/-)	20	222356	139433		115		
			WT (+/+)	20	185274	141085	Cortex, CTX (pool from two mice)	100		
			KI (+/-)	20	272220	155605		133		
		F	WT (+/+)	20	15451	16427	Brain stem, BS (pool from two mice)	100		S2d
			KI (+/-)	20	15092	16250		99		
			WT (+/+)	20	11209	19654	Hippocampus, HC (pool from two mice)	100		
			KI (+/-)	20	9668	19652		86		
			WT (+/+)	20	18859	13616	Cerebellum, CRBL (pool from two mice)	100		
			KI (+/-)	20	21175	14356		106		
			WT (+/+)	20	20131	16959	Cortex, CTX (pool from two mice)	100		
			KI (+/-)	20	18966	16806		95		
		M	WT (+/+)	20	120574	115503	Brain stem, BS (pool from two mice)	100		-
			KI (+/-)	20	103744	115737		86		
			WT (+/+)	20	96499	170680	Hippocampus, HC (pool from two mice)	100		
			KI (+/-)	20	84293	163087		91		
			WT (+/+)	20	109916	111803	Cerebellum, CRBL (pool from two mice)	100		
			KI (+/-)	20	113856	122457		95		
			WT (+/+)	20	139838	140863	Cortex, CTX (pool from two mice)	100		
			KI (+/-)	20	178021	139586		128		
		M	WT (+/+)	20	11923	12902	Brain stem, BS (pool from two mice)	100		-
			KI (+/-)	20	12720	14088		98		
			WT (+/+)	20	9303	21282	Hippocampus, HC (pool from two mice)	100		
			KI (+/-)	20	6784	21469		72		
			WT (+/+)	20	22211	12306	Cerebellum, CRBL (pool from two mice)	100		
			KI (+/-)	20	15859	10371		85		
			WT (+/+)	20	18456	15959	Cortex, CTX (pool from two mice)	100		
			KI (+/-)	20	13412	11263		103		
		M+F	WT (+/+) = 4	See above	See above	See above	Brain stem, BS (pool from two mice)	100	See above	S2f
			KI (+/-) = 4					97 ±4		
		M+F	WT (+/+) = 4				Hippocampus, HC (pool from two mice)	100		
			KI (+/-) = 4					90 ±8		
		M+F	WT (+/+) = 4				Cerebellum, CRBL (pool from two mice)	100		
			KI (+/-) = 4					100 ±7		
		M+F	WT (+/+) = 4				Cortex, CTX (pool from two mice)	100		
			KI (+/-) = 4					115 ±9		

Supplementary Table 2. Western blotting – *continued*

Test	Age (days)	Sex (F/M)	Genotype (n)	Load (µg pr lane)	α_2-protein amount (Arbitrary Units, AU)	GAPDH-protein amount (Arbitrary Units, AU)	Reagents (or indicated information)	Relative α_2-level quantification (%) (± s.e.m.)	Information	Figure
SDS-page Western blot	Adult	F	WT (+/+)	20	235821	142913	Brain stem, BS (pool from two mice)	100	Rabbit anti human α_2 aa 432-455 (07674, Upstate) Millipore Corp 1:5 000 dilution<br>Rabbit anti human GAPDH aa 1-335 (sc-25778) Santa Cruz Biotechnology Inc 1:200 dilution<br>Molecular weights: α_2-isoform:~110 kDa GAPDH:~35 kDa	-
			KI (+/-)	20	133120	135984		59		
			WT (+/+)	20	103624	153216	Hippocampus, HC (pool from two mice)	100		
			KI (+/-)	20	97514	163488		88		
			WT (+/+)	20	201348	144467	Cerebellum, CRBL (pool from two mice)	100		
			KI (+/-)	20	141156	141085		72		
			WT (+/+)	20	140766	141085	Cortex, CTX (pool from two mice)	100		
			KI (+/-)	20	136007	155605		88		
		F	WT (+/+)	20	235821	142913	Brain stem, BS (pool from two mice)	100		-
			KI (+/-)	20	133120	135984		59		
			WT (+/+)	20	93253	179697	Hippocampus, HC (pool from two mice)	100		
			KI (+/-)	20	78174	186786		79		
			WT (+/+)	20	171441	139993	Cerebellum, CRBL (pool from two mice)	100		
			KI (+/-)	20	124104	144467		70		
			WT (+/+)	20	122966	141085	Cortex, CTX (pool from two mice)	100		
			KI (+/-)	20	72263	155605		53		
		M	WT (+/+)	20	226421	127707	Brain stem, BS (pool from two mice)	100		-
			KI (+/-)	20	116729	134376		49		
			WT (+/+)	20	89199	153216	Hippocampus, HC (pool from two mice)	100		
			KI (+/-)	20	62611	163488		68		
			WT (+/+)	20	147506	124047	Cerebellum, CRBL (pool from two mice)	100		
			KI (+/-)	20	112679	134763		70		
			WT (+/+)	20	97709	168624	Cortex, CTX (pool from two mice)	100		
			KI (+/-)	20	65783	144650		78		
		M	WT (+/+)	20	244055	14027	Brain stem, BS (pool from two mice)	100		S2d
			KI (+/-)	20	124953	12995		55		
			WT (+/+)	20	80043	17595	Hippocampus, HC (pool from two mice)	100		
			KI (+/-)	20	50545	19154		58		
			WT (+/+)	20	179391	12032	Cerebellum, CRBL (pool from two mice)	100		
			KI (+/-)	20	100373	14437		47		
			WT (+/+)	20	114012	16591	Cortex, CTX (pool from two mice)	100		
			KI (+/-)	20	47975	17100		41		
		M+F	WT (+/+) = 6	See above	See above	See above	Brain stem, BS (pool from two mice)	100	See above	S2e
			KI (+/-) = 6					48 ±6		
		M+F	WT (+/+) = 6				Hippocampus, HC (pool from two mice)	100		
			KI (+/-) = 6					70 ±5		
		M+F	WT (+/+) = 6				Cerebellum, CRBL (pool from two mice)	100		
			KI (+/-) = 6					61 ±6		
		M+F	WT (+/+) = 6				Cortex, CTX (pool from two mice)	100		
			KI (+/-) = 6					67 ±7		

Supplementary Table 2. Western blotting – *continued*

Test	Age (days)	Sex (F/M)	Genotype (n)	Load (µg pr lane)	α_3-protein amount (Arbitrary Units, AU)	GAPDH-protein amount (Arbitrary Units, AU)	Reagents (or indicated information)	Relative α_3-level quantification (%) (± s.e.m.)	Information	Figure
SDS-page Western blot	Adult	F	WT (+/+)	20	150399	94140	Brain stem, BS (pool from two mice)	100	Rabbit anti rat α_3 aa 320-514 (06172, Upstate) Millipore Corp 1:1 000 dilution<br>Rabbit anti human GAPDH aa 1-335 (sc-25778) Santa Cruz Biotechnology Inc 1:200 dilution<br>Molecular weights: α_3-isoform: ~ 110 kDa GAPDH:~ 35 kDa	-
			KI (+/-)	20	198658	92460		134		
			WT (+/+)	20	111770	80556	Cerebellum, CRBL (pool from two mice)	100		
			KI (+/-)	20	147849	112936		94		
			WT (+/+)	20	139249	130709	Cortex, CTX (pool from two mice)	100		
			KI (+/-)	20	118444	122524		91		
		F	WT (+/+)	20	21665	8126	Brain stem, BS (pool from two mice)	100		-
			KI (+/-)	20	18991	13904		51		
			WT (+/+)	20	5583	13329	Hippocampus, HC (pool from two mice)	100		
			KI (+/-)	20	7889	18225		103		
			WT (+/+)	20	8604	12237	Cerebellum, CRBL (pool from two mice)	100		
			KI (+/-)	20	10736	12645		121		
			WT (+/+)	20	9424	10056	Cortex, CTX (pool from two mice)	100		
			KI (+/-)	20	13522	10056		143		
		M	WT (+/+)	20	161216	108086	Brain stem, BS (pool from two mice)	100		-
			KI (+/-)	20	216706	121813		119		
			WT (+/+)	20	41485	187226	Hippocampus, HC (pool from two mice)	100		
			KI (+/-)	20	43255	216017		90		
			WT (+/+)	20	46201	137354	Cerebellum, CRBL (pool from two mice)	100		
			KI (+/-)	20	54160	129385		124		
		M	WT (+/+)	20	10436	15449	Hippocampus, HC (pool from two mice)	100		S2d
			KI (+/-)	20	9006	21749		61		
			WT (+/+)	20	7068	14595	Cerebellum, CRBL (pool from two mice)	100		
			KI (+/-)	20	5485	15290		74		
			WT (+/+)	20	12405	22008	Cortex, CTX (pool from two mice)	100		
			KI (+/-)	20	7411	16430		80		
		F	WT (+/+)	20	6632	21403	Hippocampus, HC (pool from two mice)	100		-
			KI (+/-)	20	5146	20475		81		
			WT (+/+)	20	3527	13960	Cerebellum, CRBL (pool from two mice)	100		
			KI (+/-)	20	3178	16084		78		
			WT (+/+)	20	4048	14851	Cortex, CTX (pool from two mice)	100		
			KI (+/-)	20	3627	14960		89		
		M	WT (+/+)	20	20413	14947	Brain stem, BS (pool from two mice)	100		-
			KI (+/-)	20	19654	15100		95		
			WT (+/+)	20	7089	16555	Hippocampus, HC (pool from two mice)	100		
			KI (+/-)	20	8548	20934		95		
			WT (+/+)	20	5158	12322	Cerebellum, CRBL (pool from two mice)	100		
			KI (+/-)	20	6741	13334		121		
			WT (+/+)	20	6522	13956	Cortex, CTX (pool from two mice)	100		
			KI (+/-)	20	10558	15195		149		
		M+F	WT (+/+) = 4	See above	See above	See above	Brain stem, BS (pool from two mice)	100	See above	S2e
			KI (+/-) = 4					100 ±18		
		M+F	WT (+/+) = 5				Hippocampus, HC (pool from two mice)	100		
			KI (+/-) = 5					86 ±7		
		M+F	WT (+/+) = 6				Cerebellum, CRBL (pool from two mice)	100		
			KI (+/-) = 6					102 ±9		
		M+F	WT (+/+) = 5				Cortex, CTX (pool from two mice)	100		
			KI (+/-) = 5					110 ±15		

Supplementary Table 3. CSD Electrophysiology

Test	Age (days)	Sex (F/M)	Measurement	Genotype/ Animals (n)	Information	Mean	s.e.m.	Statistical test	Correction and/or *post hoc* test	*P*-value	Figure
Electro-physiology	175-245	M	CSD propagation speed (µm/s)	WT = 7	-	31.2	3.5	Unpaired two-tailed *t*-test	-	Genotype: $P = 0.1202$	Data not shown
				KI = 8		47.9	8.9				
Electro-physiology	175-245	M	ECoG (mV) ~ effect of CSD	WT = 7	*pre* CSD -3 to -2 min	0.043	0.004	Linear mixed models	Paired *t*-test	*Pre* vs. *post* CSD $P < 0.001$<br>WT: $P < 0.001$<br>KI: $P < 0.003$	1b
				KI = 7		0.053	0.007				
				WT = 6	1.5 min *post* CSD	0.020	0.004				
				KI = 7		0.018	0.002				
Electro-physiology	175-245	M	ECoG (% of *pre* CSD) ~ CSD recovery	WT = 7	*pre* CSD -3 to -2 min	100.00	0.00	Linear mixed models (*pre* CSD values were used for normalization)	-	Genotype: $P < 0.001$	1c 1d
				KI = 7		100.00	0.00				
				WT = 7	0-1 min *post* CSD	49.45	3.40				
				KI = 7		46.34	7.25				
				WT = 6	1-2 min *post* CSD	44.32	4.38				
				KI = 7		36.65	6.88				
				WT = 7	2-3 min *post* CSD	43.37	3.99				
				KI = 7		35.06	5.95				
				WT = 7	3-4 min *post* CSD	49.59	5.20				
				KI = 7		35.82	5.62				
				WT = 7	4-5 min *post* CSD	54.32	5.57				
				KI = 7		38.09	5.31				
				WT = 7	5-6 min *post* CSD	58.43	6.30				
				KI = 7		42.17	5.77				
				WT = 7	6-7 min *post* CSD	61.36	6.46				
				KI = 7		44.78	4.96				
				WT = 7	7-8 min *post* CSD	63.54	6.27				
				KI = 7		48.92	5.36				
				WT = 7	8-9 min *post* CSD	62.76	6.28				
				KI = 7		51.00	5.63				
				WT = 7	9-10 min *post* CSD	63.70	6.27				
				KI = 6		50.92	6.17				
				WT = 7	10-11 min *post* CSD	64.33	6.82				
				KI = 7		54.70	5.71				
				WT = 7	11-12 min *post* CSD	65.14	6.49				
				KI = 7		56.31	6.60				
				WT = 7	12-13 min *post* CSD	64.29	6.58				
				KI = 7		57.93	6.34				
				WT = 7	13-14 min *post* CSD	65.04	6.25				
				KI = 7		61.70	6.91				
				WT = 7	30 min *post* CSD	63.62	6.56				
				KI = 7		66.42	6.89				
				WT = 7	45 min *post* CSD	64.32	7.29				
				KI = 6		65.62	7.23				
				WT = 6	60 min *post* CSD	62.96	6.32				
				KI = 6		63.29	6.32				

Test	Age (days)	Sex (F/M)	Measurement	Genotype/ Animals (n)	Information	Mean	s.e.m.	Statistical test	*P*-value	Figure
Electro-physiology	174-245	M	Evoked potentials (% of *pre* CSD)	WT = 8	15 min *post* CSD	86	8	Linear mixed models	Genotype: $P = 0.2$	1e 1f
				KI = 8		89	8			

Supplementary Table 3. CSD Electrophysiology – *continued*

Test	Age (days)	Sex (F/M)	Measurement	Genotype/ Animals (n)	Information	Mean	s.e.m.	Statistical test	Correction and/or *post hoc* test	*P*-value	Figure
Laser-Doppler flowmetry	175-245	M	Cerebral blood flow (CBF) (% of *pre* CSD baseline)	WT = 8	*pre* CSD	100	0.00	Linear mixed models	Outlier correction	Genotype: $P > 0.3$	1e 1f
				KI = 7		100	0.00				
				WT = 8	5 min *post* CSD	77.40	3.45				
				KI = 7		73.70	3.40				
				WT = 8	10 min *post* CSD	75.12	2.98				
				KI = 7		73.61	2.49				
				WT = 8	15min *post* CSD	72.46	2.45				
				KI = 7		72.07	2.51				
				WT = 8	20 min *post* CSD	73.60	2.88				
				KI = 7		73.31	2.87				
				WT = 8	30 min *post* CSD	75.45	2.80				
				KI = 7		76.62	3.89				
				WT = 8	40 min *post* CSD	77.42	3.69				
				KI = 7		82.02	4.56				
				WT = 8	50 min *post* CSD	78.56	5.11				
				KI = 7		79.28	4.43				
				WT = 7	60 min *post* CSD	81.11	5.79				
				KI = 7		84.49	5.55				

Supplementary Table 4. Behavioral testing

Test	Age (days)	Sex (F/M)	Measurement	Compound (time *pre* test)	Genotype/ Animals (n)	Information	Mean	s.e.m.	Statistical test	Correction and/or *post hoc* test	*P*-value	Figure
Open field	49-100	F	Distance travelled (m)	-	WT = 13	-	24.91	2.01	Unpaired two-tailed *t*-test	-	Genotype: *P* = 0.010	2a
					KI = 18		18.69	1.26				
			Mean velocity (cm/s)		WT = 13		41.52	3.35	Unpaired two-tailed *t*-test	-	Genotype: *P* = 0.010	2b
					KI = 18		31.16	2.10				
			Faecal boli (n)		WT = 13		0.69	0.36	Unpaired two-tailed *t*-test	-	Genotype: *P* = 0.021	2c
					KI = 18		2.39	0.52				
			Urinations (n)		WT = 13		0.00	0.00	Two-sided Fisher´s exact test	-	Genotype: *P* = 0.028	2d
					KI = 18		0.39	0.14				
			Center/perimeter ratio		WT = 13		0.28	0.07	Unpaired two-tailed *t*-test	-	Genotype: *P* = 0.402	2e
					KI = 18		0.22	0.04				
	59-87	F	Distance travelled (m)	Depoprovera (20 days)	WT = 12	-	23.75	1.12	Unpaired two-tailed *t*-test	-	Genotype: *P* = 0.095	2g
					KI = 13		20.78	1.28				
			Mean velocity (cm/s)		WT = 12		39.88	1.80	Unpaired two-tailed *t*-test	-	Genotype: *P* = 0.083	2h
					KI = 13		34.86	2.07				
			Faecal boli (n)		WT = 12		1.17	0.34	Unpaired two-tailed *t*-test	-	Genotype: *P* = 0.449	2i
					KI = 13		1.62	0.46				
			Urinations (n)		WT = 12		0.00	0.00	Two-sided Fisher´s exact test	-	Genotype: *P* = 1.000	2j
					KI = 13		0.08	0.08				
			Center/perimeter ratio		WT = 12		0.27	0.06	Unpaired two-tailed *t*-test	Welch´s correction	Genotype: *P* = 0.235	2k
					KI = 13		0.18	0.03				
	43-100	M	Distance travelled (m)	-	WT = 21	-	24.18	1.45	Unpaired two-tailed *t*-test	-	Genotype: *P* = 0.213	S3a
					KI = 23		21.76	1.27				
			Mean velocity (cm/s)		WT = 21		40.31	2.41	Unpaired two-tailed *t*-test	-	Genotype: *P* = 0.207	S3b
					KI = 23		36.23	2.10				
			Faecal boli (n)		WT = 21		1.38	0.37	Unpaired two-tailed *t*-test	-	Genotype: *P* = 0.057	S3c
					KI = 23		2.52	0.44				
			Urinations (n)		WT = 21		0.05	0.05	Unpaired two-tailed *t*-test	Welch´s correction	Genotype: *P* = 0.092	S3d
					KI = 23		0.26	0.11				
			Center/perimeter ratio		WT = 21		0.17	0.02	Unpaired two-tailed *t*-test	-	Genotype: *P* = 0.562	S3e
					KI = 23		0.15	0.02				

Test	Age in days (average ± s.e.m.)	Sex (F/M)	Measurement	Compound (time *pre* test)	Genotype/ Animals (n)	Information	Mean	s.e.m.	Statistical test	*P*-value	Figure
Accelerating rotarod	174-195 (187 ±4)	F	Latency to fall (s)	-	WT = 4	Day 1	110.25	9.77	Two-way ANOVA	Genotype: *P* = 0.018	2f
						Day 2	131.50	18.13			
						Day 3	133.88	18.62			
	174-195 (184 ±4)				KI = 5	Day 1	94.10	6.34			
						Day 2	98.90	10.42			
						Day 3	106.80	11.40			
	184-226 (207 ±6)			Depoprovera (21-28 days)	WT = 8	Day 1	102.50	9.21	Two-way ANOVA	Genotype: *P* = 0.132	2l
						Day 2	105.13	7.34			
						Day 3	123.06	9.22			
	111-217 (178 ±13)				KI = 9	Day 1	105.39	8.16			
						Day 2	120.22	8.27			
						Day 3	136.00	7.56			

Test	Age (days)	Sex (F/M)	Measurement	Genotype/ Animals (n)	Mean	s.e.m.	Statistical test	Correction and/or *post hoc* test	*P*-value	Figure
Grip strength	51-102	F	Grip strength/body weight	WT = 13	8.28	0.32	Unpaired two-tailed *t*-test	Welch´s correction	Genotype: *P* = 0.126	S4a
				KI = 17	7.71	0.14				
	45-102	M		WT = 22	7.14	0.26	Unpaired two-tailed *t*-test	Welch´s correction	Genotype: *P* = 0.131	S4b
				KI = 23	6.69	0.14				

Supplementary Table 4. Behavioral testing – *continued*

Test	Age (days)	Sex (F/M)*	Measurement	Compound (time *pre* test)	Genotype/ Animals (n)	Information	Mean	s.e.m.	Statistical test	Correction and/or *post hoc* test	*P*-value	Figure
Acoustic startle response, ASR	59-98	(2/6) *P*=0.863	ASR (%)	-	WT = 8	None	11.29	1.82	Two-way ANOVA	-	Genotype: *P* < 0.0001	4a 4d
						80 dB	10.64	1.15				
						90 dB	14.85	2.44				
						100 dB	27.23	2.84				
						105 dB	35.46	2.64				
						110 dB	41.98	3.94				
						120 dB	40.32	4.54				
	59-98	(5/4) *P*=0.805		-	KI = 9	None	8.16	0.64				
						80 dB	10.78	2.19				
						90 dB	20.75	3.84				
						100 dB	38.74	5.08				
						105 dB	52.63	3.63				
						110 dB	55.67	5.50				
						120 dB	61.12	5.99				
	78-103	(4/3) *P*=0.886		Amantadine, AMA (30 min)	WT = 7	None	9.39	1.15	Two-way ANOVA	-	Genotype: *P* = 0.814	4b 4d
						80 dB	18.16	4.71				
						90 dB	24.54	7.13				
						100 dB	25.17	4.55				
						105 dB	39.69	4.49				
						110 dB	37.12	4.98				
						120 dB	43.04	3.54				
	78-103	(5/2) *P*=0.096			KI = 7	None	10.57	1.82				
						80 dB	11.58	2.42				
						90 dB	16.67	3.51				
						100 dB	32.60	6.52				
						105 dB	38.00	4.09				
						110 dB	38.49	2.05				
						120 dB	41.72	4.72				
	65-93	(7/5) *P*=0.338		PBS (30 min)	WT = 12	None	8.05	0.59	Two-way ANOVA	-	Genotype: *P* = 0.0181	4c 4d
						80 dB	7.60	1.07				
						90 dB	10.28	1.38				
						100 dB	20.39	2.35				
						105 dB	28.62	3.58				
						110 dB	28.94	3.82				
						120 dB	36.02	4.32				
	78-93	(3/2) *P*=0.695		PBS (30 min)	KI = 5	None	6.56	0.41				
						80 dB	5.39	0.38				
						90 dB	6.80	0.56				
						100 dB	20.11	5.15				
						105 dB	36.20	9.49				
						110 dB	37.61	5.55				
						120 dB	62.01	6.59				
	78-93	See above		PBS	WT = 12	120 dB	36.02	4.32	Unpaired two-tailed *t*-test	Welch´s correction	Genotype: *P* = 0.0132	
	78-93			PBS	KI = 5	120 dB	62.01	6.59				
	59-98			None	WT = 8	120 dB	40.32	4.54	Unpaired two-tailed *t*-test	-	None/AMA: *P* = 0.651 None/PBS: *P* = 0.515 AMA/PBS: *P* = 0.282	4d
	78-103			AMA	WT = 7	120 dB	43.04	3.54				
	78-93			PBS	WT = 12	120 dB	36.02	4.32				

*All male and female data sets were statistically comparable (P > 0.05) and thus pooled. Test for pool of male and female data was carried out using two-way ANOVA.

Supplementary Table 4. Behavioral testing – *continued*

Test	Age (days)	Sex (F/M)	Measurement	Compound (time *pre* test)	Genotype/ Animals (n)	Information	Mean	s.e.m.	Statistical test	Correction and/or *post hoc* test	*P*-value	Figure
Open field	49-100	F	Groomings (n)	-	WT = 12	-	1.67	0.28	Unpaired two-tailed *t*-test	Outlier correction	Genotype: *P* = 0.024	4e
					KI = 18		2.78	0.33				
	59-87	F		Depoprovera (20 days)	WT = 12		1.17	0.21	Unpaired two-tailed *t*-test	-	Genotype: *P* = 0.839	
					KI = 13		1.23	0.23				
	43-100	M		-	WT = 21		2.57	0.30	Unpaired two-tailed *t*-test	-	Genotype: *P* = 0.638	
					KI = 23		2.39	0.24				
	49-100	F		-	WT = 12		1.67	0.28	Unpaired two-tailed *t*-test	Outlier correction	Treatment: *P* = 0.169	
	59-87	F		Depoprovera	WT = 12		1.17	0.21				
	49-100	F		-	KI = 18		2.78	0.33	Unpaired two-tailed *t*-test	-	Treatment: *P* = 0.0013	
	59-85	F		Depoprovera	KI = 13		1.23	0.23				
Marble burying	41-106	F	Marbles buried (n)	-	WT = 25	-	8.52	1.12	Unpaired two-tailed *t*-test	-	Genotype: *P* = 0.002	4f 4i
					KI = 23		14.09	1.29	One-way ANOVA	Tukey´s *post hoc*	Genotype: *P* < 0.05, ★★	
	41-104	M		-	WT = 31		13.84	1.11	Unpaired two-tailed *t*-test	-	Genotype: *P* = 0.184	
					KI = 14		11.00	1.96				
	84-200	F		Amantadine (30 min)	WT = 11		2.91	0.74	Unpaired two-tailed *t*-test	Welch´s correction	Genotype: *P* = 0.626	4g
					KI = 9		4.11	2.26	One-way ANOVA	Tukey´s *post hoc*	Genotype: *P* > 0.05, ns	
	84-195	F		PBS (30 min)	WT = 11		8.73	1.61	Unpaired two-tailed *t*-test	-	Genotype: *P* = 0.0004	
					KI = 8		19.38	1.81	One-way ANOVA	Tukey´s *post hoc*	Genotype:*P*<0.05, ★★★	
	62-196	F		Depoprovera (18-33 days)	WT = 18		9.44	1.34	Unpaired two-tailed *t*-test	-	Genotype: *P* = 0.702	4i
					KI = 20		8.70	1.37	One-way ANOVA	Tukey´s *post hoc*	Genotype: *P* > 0.05, ns	
	See above	F		PBS vs Amantadine	WT = 11	PBS	8.73	1.61	One-way ANOVA	Tukey´s *post hoc*	Genotype: *P* > 0.05, ns	4g
					WT = 11	Amantadine	2.91	0.74				
	See above	F		PBS vs Amantadine	KI = 8	PBS	19.38	1.81	One-way ANOVA	Tukey´s *post hoc*	Genotype:*P*<0.05, ★★★	
					KI = 9	Amantadine	4.11	2.26				
	See above	F		None vs Depoprovera	WT = 25	None	8.52	1.12	One-way ANOVA	Tukey´s *post hoc*	Genotype: *P* > 0.05, ns	4i
					WT = 18	Depoprovera	9.44	1.34				
	See above	F		None vs Depoprovera	KI = 23	None	14.09	1.29	One-way ANOVA	Tukey´s *post hoc*	Genotype: *P* < 0.05,	
					KI = 20	Depoprovera	8.70	1.37				
	50-81	F	Marbles buried (n)	Memantine (30 min)	WT = 11	-	13.27	1.46	Unpaired two-tailed *t*-test	-	Genotype: *P* = 0.776	4h
					KI = 10		12.70	1.32	One-way ANOVA	Tukey´s *post hoc*	Genotype: *P* > 0.05, ns	
	41-76	F		PBS (30 min)	WT = 15		12.80	1.02	Unpaired two-tailed *t*-test	Welch´s correction	Genotype: *P* = 0.0051	
					KI = 10		19.50	1.11	One-way ANOVA	Tukey´s *post hoc*	Genotype: *P* < 0.05, ★	
	See above	F		PBS vs Memantine	WT = 15	PBS	12.80	1.02	Unpaired two-tailed *t*-test	-	Genotype: *P* = 0.725	
					WT = 11	Memantine	13.27	1.46	One-way ANOVA	Tukey´s *post hoc*	Genotype: *P* > 0.05, ns	
	See above	F		PBS vs Memantine	KI = 10	PBS	19.50	1.11	Unpaired two-tailed *t*-test	-	Genotype: *P* = 0.01	
					KI = 10	Memantine	12.70	1.32	One-way ANOVA	Tukey´s *post hoc*	Genotype: *P* > 0.05, ns	

Test	Age (days)	Sex (F/M)*	Measurement	Genotype/ Animals (n)	Information	Mean	s.e.m.	Statistical test	Correction and/or *post hoc* test	*P*-value	Figure
Elevated plus maze, EPM	57-65	(5/5)	Distance (cm)	WT = 10	-	14.77	1.05	Unpaired two-tailed *t*-test	-	Genotype: *P* = 0.739	S7a
	59-65	(5/5)		KI = 10		15.30	1.17				
	57-65	(5/5)	Open arm time (%)	WT = 10		6.90	1.58	Unpaired two-tailed *t*-test	-	Genotype: *P* = 0.878	S7b
	59-65	(5/5)		KI = 10		7.24	1.47				
	57-65	(5/5)	Open arm entries (%)	WT = 10		29.71	2.88	Unpaired two-tailed *t*-test	-	Genotype: *P* = 0.337	S7c
	59-65	(5/5)		KI = 10		25.77	2.77				
	57-65	(5/5)	Line crossings (n)	WT = 10		112.10	7.16	Unpaired two-tailed *t*-test	-	Genotype: *P* = 0.556	S7d
	59-65	(5/5)		KI = 10		104.80	9.83				
	95-110	(5/5)	Distance (cm)	WT = 10	Stress *pre* EPM	13.17	1.11	Unpaired two-tailed *t*-test	-	Genotype: *P* = 0.312	S7e
	106-110	(5/5)		KI = 10		11.77	0.76				
	95-110	(5/5) -	Open arm time (%)	WT = 10		4.43	1.63	Unpaired two-tailed *t*-test	-	Genotype: *P* = 0.626	S7f
	106-110	(5/5) -		KI = 10		3.39	1.33				
	95-110	(5/5)	Open arm entries (%)	WT = 10		20.56	1.66	Unpaired two-tailed *t*-test	Welch´s correction	Genotype: *P* = 0.437	S7g
	106-110	(5/5)		KI = 10		17.45	3.50				

*All male and female data sets were statistically comparable (P > 0.05) and thus pooled. Test for pool of male and female data was carried out using one-way ANOVA with Tukey´s post hoc comparison test (data sets with same variance (Bartlett´s test) or Kruskal-Wallis with Dunn´s post hoc comparison test (data sets with unequal variance (Bartlett´s test); the latter is denoted with ★.

Supplementary Table 4. Behavioral testing – *continued*

Test	Age (days)	Sex (F/M)	Measurement	Genotype/ Animals (n)	Information	Mean	s.e.m.	Statistical test	Correction and/or *post hoc* test	*P*-value	Figure
Three-chamber social preference	85-100	F	Chamber time (s)	WT = 5	Stranger, S	161.00	14.88	One-way ANOVA	Tukey´s *post hoc*	S vs E: $P < 0.05$, ⋆⋆⋆	5a
					Center,C	162.10	20.14				
					Empty, E	61.18	17.26				
	96-100	F		KI = 5	Stranger, S	138.10	12.28	One-way ANOVA	Tukey´s *post hoc*	S vs E: $P > 0.05$, ns	
					Center, C	119.90	18.09				
					Empty, E	100.10	8.53				
	85-100	M		WT = 5	Stranger, S	176.30	18.56	One-way ANOVA	Tukey´s *post hoc*	S vs E: $P < 0.05$, ⋆⋆	
					Center, C	90.98	16.84				
					Empty, E	98.88	16.09				
	85-100	M		KI = 5	Stranger, S	126.00	8.39	One-way ANOVA	Tukey´s *post hoc*	S vs E: $P > 0.05$, ns	
					Center, C	149.70	37.05				
					Empty, E	97.74	6.07				
	85-100	F	Sniffing time (s)	WT = 5	Stranger, S	157.70	17.91	One-way ANOVA	Tukey´s *post hoc*	S vs E: $P < 0.05$, ⋆⋆	5b
					Empty, E	63.84	14.86				
	96-100	F		KI = 5	Stranger, S	145.90	7.38	One-way ANOVA	Tukey´s *post hoc*	S vs E: $P > 0.05$, ns	
					Empty, E	95.52	10.64				
	85-100	M		WT = 5	Stranger, S	167.30	24.59	One-way ANOVA	Tukey´s *post hoc*	S vs E: $P < 0.05$, ⋆⋆	
					Empty, E	73.58	13.47				
	85-100	M		KI = 5	Stranger, S	133.30	27.05	One-way ANOVA	Tukey´s *post hoc*	S vs E: $P > 0.05$, ns	
					Empty, E	84.74	7.96				
	85-100	F	Chamber entries (n)	WT = 5	Stranger, S	67.20	3.76	One-way ANOVA	Tukey´s *post hoc*	S vs E: $P < 0.05$, ⋆⋆	5c
					Center, C	13.00	2.30				
					Empty, E	28.00	5.41				
	96-100	F		KI = 5	Stranger, S	72.80	6.28	One-way ANOVA	Tukey´s *post hoc*	S vs E: $P > 0.05$, ns	
					Center, C	24.40	2.40				
					Empty, E	52.20	3.06				
	85-100	M		WT = 5	Stranger, S	84.60	5.01	One-way ANOVA	Tukey´s *post hoc*	S vs E: $P < 0.05$, ⋆⋆	
					Center, C	20.60	1.12				
					Empty, E	46.40	9.68				
	85-100	M		KI = 5	Stranger, S	61.60	9.79	One-way ANOVA	Tukey´s *post hoc*	S vs E: $P > 0.05$, ns	
					Center, C	19.40	3.11				
					Empty, E	36.60	4.23				

Supplementary Table 4. Behavioral testing – *continued*

Test		Age (days)	Sex (F/M)*	Measurement	Genotype/ Animals	Information	Mean	s.e.m.	Statistical test	Correction and/or *post hoc* test	*P*-value	Figure
Tail suspension (TST)		75-194	(4/17) -	Immobility (s)	WT = 21	0-2 min	22.59	3.17	Two-way ANOVA	-	Genotype: *P* = 0.0033	6a
						2-4 min	42.89	2.98				
						4-6 min	45.53	5.08				
		59-194	(9/7) -		KI = 16	0-2 min	29.96	3.90				
						2-4 min	56.73	6.33				
						4-6 min	59.38	6.84				
		75-194	(4/17) -	Absolute turn angles (degrees)	WT = 21	0-2 min	48 142	1 864	Two-way ANOVA	-	Genotype: *P* < 0.0001	6b
						2-4 min	33 526	2 042				
						4-6 min	30 922	3 072				
		59-194	(9/7) -		KI = 16	0-2 min	40 554	2 333				
						2-4 min	20 327	2 855				
						4-6 min	17 242	2 756				
		75-194	(4/17) -	Rotations (n)	WT = 21	0-2 min	2.80	0.64	Two-way ANOVA	Outlier correction§	Genotype: *P* < 0.0001	6c
						2-4 min	2.19	0.67				
						4-6 min	1.29	0.35				
		59-194	(9/7) -		KI = 16	0-2 min	0.67	0.46				
						2-4 min	0.07	0.07				
						4-6 min	0.00	0.00				
		75-194	(4/17) -	Mobility (s)	WT = 21	0-2 min	97.42	3.17	Two-way ANOVA	-	Genotype: *P* = 0.0014	6d
						2-4 min	77.11	2.98				
						4-6 min	73.34	5.17				
		59-194	(9/7) -		KI = 16	0-2 min	90.06	3.90				
						2-4 min	63.29	6.33				
						4-6 min	55.88	6.92				
		75-194	(4/17)	Vocalization (%)	WT = 21	6 min	14.29	7.83	Unpaired two-tailed *t*-test	-	Genotype: *P* = 0.0179	6e
		59-194	(9/7)		KI = 16	6 min	50.00	12.91				

*All male and female data sets were statistically comparable (P > 0.05) and thus pooled. Test for pool of male and female data was carried out using one-way ANOVA with Tukey´s post hoc comparison test (data sets with same variance (Bartlett´s test) or Kruskal-Wallis with Dunn´s post hoc comparison test (data sets with unequal variance (Bartlett´s test); the latter is denoted with ⋆.

Test	Age (days)	Sex (F/M)*	Cages (n)	Measurement	Genotype/ Animals (n)	Information	Mean	s.e.m.	Statistical test	Correction and/or *post hoc* test	*P*-values	Figure
Stress-induced sucrose preference	158-213	(6/7)	n = 5	Sucrose intake (%)	WT = 13	-	97.59	0.46	Unpaired two-tailed *t*-test	-	Genotype: *P* = 0.796	6f
	158-213	(7/8)	n = 6		KI = 15		97.38	0.59				
	158-213	(6/7)	n = 5		WT = 13	Stress I	97.78	0.40	Unpaired two-tailed *t*-test	-	WT vs KI: *P* = 0.368 WT vs KI-a: *P* = 0.045	
	158-213	(4/3)	n = 2		KI = 7		95.96	0.92				
	158-213	(3/5)	n = 4		KI-a = 8		92.98	1.70				
	158-213	(3/7)	n = 4		WT = 10	Stress II	96.82	0.32	Unpaired two-tailed *t*-test	-	WT vs KI-a: *P* = 0.0012	
	158-213	(3/0)	n = 1		WT-a = 3		88.96	0.00				
	158-213	(0/3)	n = 1		KI = 3		97.31	0.00				
	158-213	(7/5)	n = 5		KI-a = 12		91.28	0.90				
	158-213	(6/7)	n = 5		WT = 13	-	97.59	0.46	Unpaired two-tailed *t*-test	-	None vs SI: *P* = 0.217 None vs S2: *P* = 0.230	
	158-213	(6/7)	n = 5		WT = 13	Stress I	97.78	0.40				
	158-213	(3/7)	n = 4		WT = 10	Stress II	96.82	0.32				
	158-213	(6/7)	n = 5	Liquid/mouse/day (g)	WT = 13	-	13.78	2.79	Unpaired two-tailed *t*-test	-	Genotype: *P* = 0.265	6g
	158-213	(7/8)	n = 6		KI = 15		10.23	1.44				
	158-213	(6/7)	n = 5		WT = 13	Stress I	17.23	1.64	Unpaired two-tailed *t*-test	-	WT vs KI: *P* = 0.332 WT vs KI-a: *P* = 0.011	
	158-213	(4/3)	n = 2		KI = 7		13.82	3.04				
	158-213	(3/5)	n = 4		KI-a = 8		10.40	0.77				
	158-213	(3/7)	n = 4		WT = 10	Stress II	18.15	1.56	Unpaired two-tailed *t*-test	-	WT vs KI-a: *P* = 0.001	
	158-213	(3/0)	n = 1		WT-a = 3		7.74	0.00				
	158-213	(0/3)	n = 1		KI = 3		12.07	0.00				
	158-213	(7/5)	n = 5		KI-a = 12		9.57	0.74				
	158-213	(6/7)	n = 5		WT = 13	-	13.78	2.79	Unpaired two-tailed *t*-test	-	None vs SI: *P* = 0.318 None vs S2: *P* = 0.246	
	158-213	(6/7)	n = 5		WT = 13	Stress I	17.23	1.64				
	158-213	(3/7)	n = 4		WT = 10	Stress II	18.15	1.56				
	158-213	(6/7)	n = 5		WT = 13	-	13.78	2.79	Unpaired two-tailed *t*-test	Welch´s correction	WT vs KI-a SI: *P*= 0.308 WT vs KI-a SII:	
	158-213	(3/5)	n = 4		KI-a = 8	Stress I	10.40	0.77				
	158-213	(7/5)	n = 5		KI-a = 12	Stress II	9.57	0.74				

*All male and female data sets were statistically comparable (P > 0.05) and thus pooled. Test for pool of male and female data was carried out using one-way ANOVA with Tukey´s post hoc comparison test (data sets with same variance (Bartlett´s test) or Kruskal-Wallis with Dunn´s post hoc comparison test (data sets with unequal variance (Bartlett´s test); the latter is denoted with ⋆.

Supplementary Table 5. Etradiol and glutamate assays, and [^{3}H] aspartate uptake

Test	Age (days)	Sex (F/M)	Measurement	Genotype/ Animals (n)	Information	Mean	s.e.m.	Statistical test	Correction and/or *post hoc* test	*P*-value	Figure
Estradiol	48-123	F	Estradiol (pg/mL)	WT = 8	Untreated	6.68	0.96	Unpaired two-tailed *t*-test (relative to no treatment)	-	-	S5
				WT = 10	Depoprovera, P8	6.47	0.57			Treatment: *P* = 0.841	
				WT = 14	Depoprovera, P18	4.95	0.46			Treatment: *P* = 0.079	
	114	M		WT = 3	Untreated	4.50	1.40	-	-	-	

Test	Age (days)	Sex (F/M)	Measurement	Compound (time *pre* test)	Genotype/ Animals (n)	Information	Mean	s.e.m.	Statistical test	Correction and/or *post hoc* test	*P*-values	Figure
Glutamate assay	128-242	F	Glutamate (nmoles/20 µg protein)	-	WT = 2 × 5	CTX	3.40	0.12	Unpaired two-tailed *t*-test	-	Genotype: *P* = 0.009	3a
					KI = 2 × 5		3.99	0.16				
					WT = 2 × 5	HC	3.27	0.24	Unpaired two-tailed *t*-test	-	Genotype: *P* = 0.026	3b
					KI = 2 × 5		4.05	0.21				
					WT = 2 × 5	CRBL	2.67	0.12	Unpaired two-tailed *t*-test	-	Genotype: *P* = 0.0007	3c
					KI = 2 × 5		3.42	0.12				
	196-233	F		Depoprovera (28 days)	WT = 1 × 5	CTX	2.88	0.18	Unpaired two-tailed *t*-test	-	Genotype: *P* = 0.455<br>+/- DP, WT: *P* = 0.032<br>+/- DP, KI: *P* = 0.0003	3a
					KI = 1 × 5		2.73	0.07				
					WT = 1 × 5	HC	2.82	0.08	Unpaired two-tailed *t*-test	-	Genotype: *P* = 0.056	3b
					KI = 1 × 5		2.59	0.04		Welch´s correction	+/- DP, WT: *P* = 0.112<br>+/- DP, KI: *P* = 0.0002	
					WT = 1 × 5	CRBL	2.40	0.06	Unpaired two-tailed *t*-test	-	Genotype: *P* = 0.942<br>+/- DP, WT: *P* = 0.169<br>+/- DP, KI: *P* = 0.0004	3c
					KI = 1 × 5		2.39	0.12				

Test	Age (days)	*In vitro* cultures	Genotype	Load (µg pr lane)	α_2-protein amount (Arbitrary Units, AU)	Actin-protein amount (Arbitrary Units, AU)	Reagents (or indicated information)	Relative α_2-level quantification	Information	Figure
SDS-page Western blot	E17	Mixed culture of astrocytes and neurons (passage 1)	WT (+/+)	100	61115	645	Rabbit anti-human α (07674, Upstate): 1:3 000 dilution Mouse anti-chicken actin (612656, BD): 1:3 000 dilution	100	α_2~ 110 kDa<br>actin:~ 42 kDa	-
			KI (+/-)	100	17984	581		33		
			KI (-/-)	100	467	569<br>33		<1		

Test	Age (days)	*In vitro* cultures	Genotype	Samples (n)	Measure	Mean*	s.e.m.	Statistical test	Correction and/or *post hoc* test	*P*-values	Figure
Glutamate uptake	E17	Mixed culture of astrocytes and neurons (passage 1)	WT (+/+)	n = 22	[^{3}H]-D-aspartate uptake (% of WT)	100.0	2.9	One-way ANOVA			3d
			KI (+/-)	n = 23		89.6	2.		Tukey´s *post hoc*	WT: *P* > 0.05, ns	
			KI (-/-)	n = 13		83.4	5.<br>3		Tukey´s *post hoc*	WT: *P* < 0.05, ⋆⋆	

*Absolutte numbers below

[^{3}H]-D-aspartate uptake (absolutte values)
(Numbers sequencially, seperated by (/))

Genotype	Counts per minute (CPM)	Protein ug/ul	CPM/Protein	Days in culture (mean +/- SD)
WT (n=22)	10201/9254/6729/7449/8363/9543/7826/6223/6743/6417/5732/5729/5296/6284/7719/7640/6405/1718/3286/1343/5006/2328	0,517651573/0,51315914/0,454908643/0,434104894/0,413301146/0,461843226/0,371693648/0,144759507/0,146018285/0,166788128/0,184411024/0,164270571/0,185669803/0,179375911/0,185669803/0,173082019/1,31/0,46/1,06/0,49/1,17/0,71	19706,30541/18015,8537/14791,98098/17159,44716/20234,64026/20662,85583/21054,97377/42988,54096/46179,14791/38473,96147/31082,74043/34875,38859/28523,75522/35032,5747/41573,8041/44140,92249/4889,312977/3734,782609/3100/2740,816327/4278,632479/3278,873239	23,86+/-1.36
KI (+/-) (n=23)	8078/2243/7390/8614/7694/6986/7454/7123/7879/2099/5653/5664/6770/4527/4260/6982/6833/4264/3903/2890/2952/8078/2243	0,421047583/0,122122026/0,431331061/0,545058222/0,411914229/0,511772224/0,470164726/0,420235728/0,542284389/0,053498079/0,159235458/0,137206837/0,171193852/0,162382404/0,125877832/1,52/1,22/1,51/1,41/0,9/1,03/0,421047583/0,122122026	19185,48006/18366,8751/17133,01142/15803,81627/18678,64584/13650,60407/15854,01794/16950,01048/14529,27682/39235,05387/35500,88704/41280,74167/39545,81269/27878,63647/33842,33684/4593,421053/5600,819672/2823,84106/2768,085106/3211,111111/2866,019417/19185,48006/18366,8751	23.65+/-1.30
KI (-/-) (n=13)	4881/6754/7264/7327/5792/6574/6119/5570/3338/2740/632/2864/4881	0,521297007/0,39370683/0,511772224/0,539510556/0,17245263/0,168676295/0,156088512/0,145388896/0,62/0,94/0,34/1,26/0,521297007	9363,184395/17154,89669/14193,81448/13580,82789/33586,03458/38974,0597/39202,1163/38311,04124/5383,870968/2914,893617/1858,823529/2273,015873/9363,184395	23.38+/-1.56

Supplementary Table 6. HPLC measurements

Test	Age (days)	Sex (F/M)*	Measurement	Genotype Animals (n)	Information	Mean	s.e.m.	Statistical test	Correction and/or *post hoc* test	*P*-value	Figure
HPLC	51-108	(9/9)	Dopamine, DA (pmoles/mg wet tissue)	WT = 18	Frontal cortex, Frontal CTX	1.06	0.15	Unpaired two-tailed *t*-test	-	Genotype: *P* = 0.384	S6a
		(8/9)		KI = 17		1.27	0.19				
		(9/9) ⋆		WT = 18	Occipital cortex, Occipital CTX	0.98	0.26	Unpaired two-tailed *t*-test	-	Genotype: *P* = 0.578	
		(8/9) ⋆		KI = 17		0.80	0.19				
		(9/9)		WT = 18	Striatum, STR	59.51	8.29	Unpaired two-tailed *t*-test	-	Genotype: *P* = 0.454	
		(8/9)		KI = 17		67.63	6.65				
		(7/9)		WT = 16	Hippocampus, HC	0.46	0.14	Unpaired two-tailed *t*-test	Welch´s correction	Genotype: *P* = 0.437	
		(8/8)		KI = 16		0.69	0.26				
		(4/2)		WT = 6	Cerebellum, CRBL	0.10	0.02	Unpaired two-tailed *t*-test	-	Genotype: *P* = 0.860	
		(3/2)		KI = 5		0.10	0.02				
		(9/9)		WT = 18	Brain stem, BS	0.62	0.04	Unpaired two-tailed *t*-test	-	Genotype: *P* = 0.084	
		(8/9)		KI = 17		0.70	0.03				
	51-108	(9/9)	Serotonin, 5-HT (pmoles/mg wet tissue)	WT = 18	Frontal cortex, Frontal CTX	2.41	0.15	Unpaired two-tailed *t*-test	-	Genotype: *P* = 0.780	S6b
		(8/9)		KI = 17		2.35	0.18				
		(9/9)		WT = 18	Occipital cortex, Occipital CTX	2.03	0.14	Unpaired two-tailed *t*-test	-	Genotype: *P* = 0.721	
		(8/9)		KI = 17		1.97	0.09				
		(9/9) ⋆		WT = 18	Striatum, STR	2.44	0.09	Unpaired two-tailed *t*-test	Welch´s correction	Genotype: *P* = 0.281	
		(8/9) ⋆		KI = 17		2.70	0.22				
		(9/9) ⋆		WT = 18	Hippocampus, HC	3.25	0.22	Unpaired two-tailed *t*-test	-	Genotype: *P* = 0.482	
		(8/9) ⋆		KI = 17		3.06	0.15				
		(9/8) ⋆		WT = 17	Cerebellum, CRBL	0.83	0.10	Unpaired two-tailed *t*-test	Welch´s correction	Genotype: *P* = 0.764	
		(8/8) ⋆		KI = 16		0.79	0.05				
		(9/9) ⋆		WT = 18	Brain stem, BS	4.22	0.31	Unpaired two-tailed *t*-test	-	Genotype: *P* = 0.233	
		(8/9) ⋆		KI = 17		4.71	0.25				
	51-108	(9/9)	Noradrenaline, NA (pmoles/mg wet tissue)	WT = 18	Frontal cortex, Frontal CTX	2.38	0.14	Unpaired two-tailed *t*-test	-	Genotype: *P* = 0.410	S6c
		(8/9)		KI = 17		2.20	0.16				
		(9/9)		WT = 18	Occipital cortex, Occipital CTX	1.74	0.11	Unpaired two-tailed *t*-test	-	Genotype: *P* = 0.931	
		(8/9)		KI = 17		1.72	0.13				
		(9/9)		WT = 18	Striatum, STR	1.12	0.11	Unpaired two-tailed *t*-test	-	Genotype: *P* = 0.545	
		(8/9)		KI = 17		1.02	0.11				
		(9/9) ⋆		WT = 18	Hippocampus, HC	2.21	0.07	Unpaired two-tailed *t*-test	Welch´s correction	Genotype: *P* = 0.277	
		(8/9) ⋆		KI = 17		2.40	0.16				
		(9/9)		WT = 18	Cerebellum, CRBL	2.39	0.12	Unpaired two-tailed *t*-test	-	Genotype: *P* = 0.447	
		(8/9)		KI = 17		2.52	0.13				
		(9/9)		WT = 18	Brain stem, BS	4.11	0.15	Unpaired two-tailed *t*-test	-	Genotype: *P* = 0.713	
		(8/9)		KI = 17		4.20	0.21				
	51-108	(9/9)	Gamma-aminobutyric acid, GABA (nmoles/mg wet tissue)	WT = 18	Frontal cortex, Frontal CTX	2.65	0.17	Unpaired two-tailed *t*-test	-	Genotype: *P* = 0.841	S6d
		(8/9)		KI = 17		2.70	0.18				
		(9/9) ⋆		WT = 18	Occipital cortex, Occipital CTX	2.39	0.13	Unpaired two-tailed *t*-test	Welch´s correction	Genotype: *P* = 0.174	
		(8/9) ⋆		KI = 17		2.81	0.27				
		(9/9) ⋆		WT = 18	Striatum, STR	3.24	0.16	Unpaired two-tailed *t*-test	Welch´s correction	Genotype: *P* = 0.296	
		(8/9) ⋆		KI = 17		3.81	0.51				
		(9/9)		WT = 18	Hippocampus, HC	4.55	0.26	Unpaired two-tailed *t*-test	-	Genotype: *P* = 0.862	
		(8/9)		KI = 17		4.49	0.23				
		(9/9) ⋆		WT = 18	Cerebellum, CRBL	2.61	0.17	Unpaired two-tailed *t*-test	-	Genotype: *P* = 0.756	
		(8/9) ⋆		KI = 17		2.69	0.19				
		(8/9)		WT = 17	Brain stem, BS	5.47	0.59	Unpaired two-tailed *t*-test	-	Genotype: *P* = 0.694	
		(8/9)		KI = 17		5.84	0.73				

*All male and female data sets were statistically comparable (P > 0.05) and thus pooled. Test for pool of male and female data was carried out using one-way ANOVA with Tukey´s post hoc comparison test (data sets with same variance (Bartlett´s test) or Kruskal-Wallis with Dunn´s post hoc comparison test (data sets with unequal variance (Bartlett´s test); the latter is denoted with ⋆.

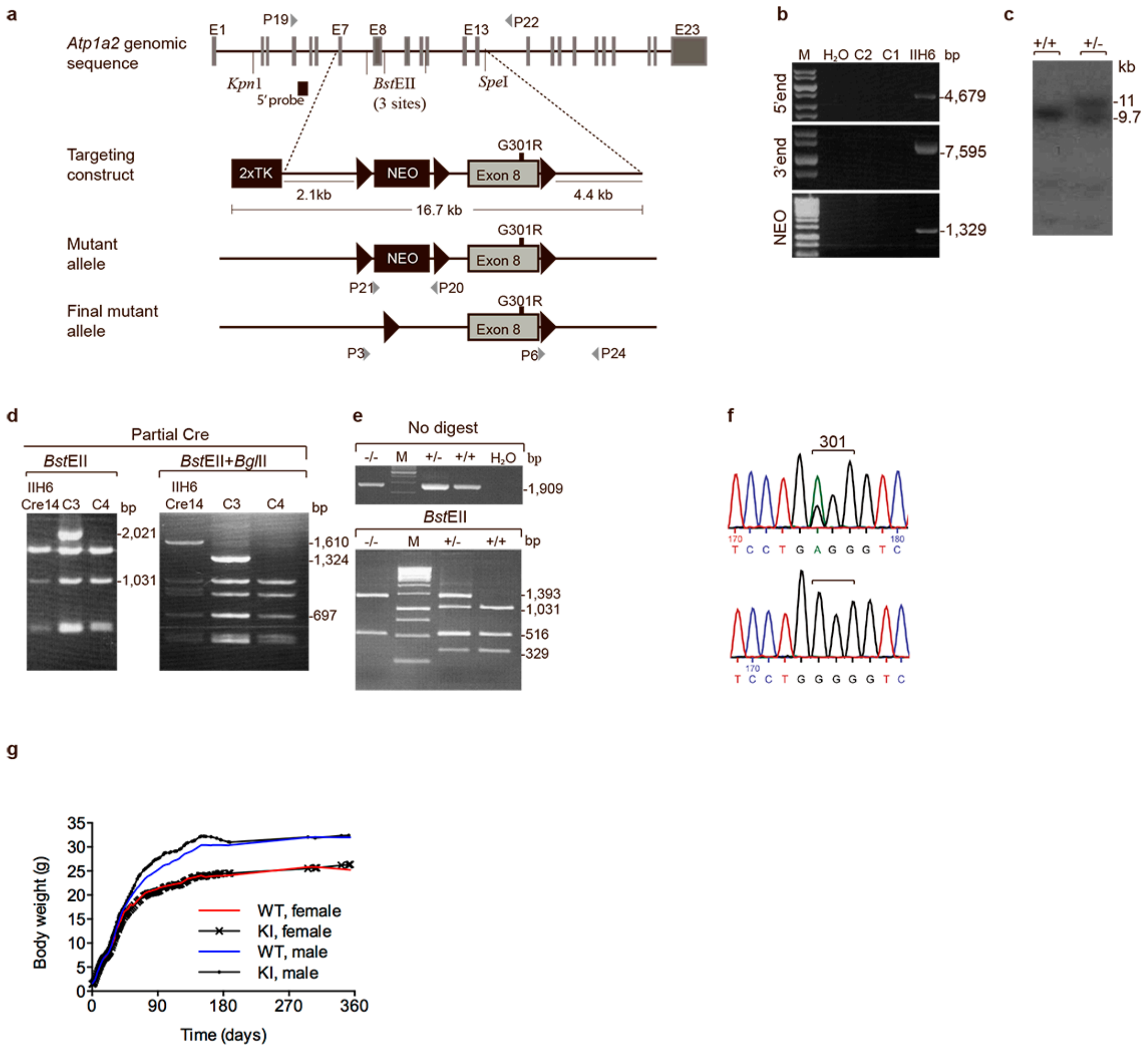

Figure S1. Targeting Strategy Screenings and Basic Characterization of the $\alpha_2^{+/G301R}$ Knock-in Mice
(**a**) Diagram of the targeting strategy showing the oligonucleotide primers (grey triangles: P3, P6, P19-P22, and P24), the 5' Southern probe (black square), and the restriction enzyme sites used for screenings and genotyping. Introduction of a 3´ LoxP site (dark triangle flanking Exon 8) abolished an endogenous *Bst*EII site. Partial Cre-excision removed the NEO cassette and left a LoxP site in intron 7. (**b**) PCR screenings verified homologous recombination in the IIH6 clone, which was not observed for two negative controls (C1 and C2). (**c**) Southern blotting analysis verified heterozygous homologous recombination in clone IIH6 not observed for control (ES cells). (**d**) Partial Cre-excision of LoxP-NEO-LoxP cassette in the IIH6Cre14 clone was verified by PCR and not observed for two controls (C3 and C4). (**e**) Crossing of two $\alpha_2^{+/G301R}$ mice gained homozygous (-/-), heterozygous (+/-) and WT (+/+) newborn pups identified by PCR-based genotyping. (**f**) Sequencing confirmed a heterozygous G→A base exchange in position 901 in the genomic DNA of a $\alpha_2^{+/G301R}$ mouse which was not present in WT. (**g**) Body weights (g) of $\alpha_2^{+/G301R}$ (females n=11 and males n=6) and WT (females n=7 and males n=7) mice at the indicated ages (days) were determined. Data shown as means, with s.e.m. omitted for clarity.
For additional informations, see Supplementary Table 1.
M; molecular DNA marker, bp; base pair, H_2O; No template control where DNA was substituted with water.

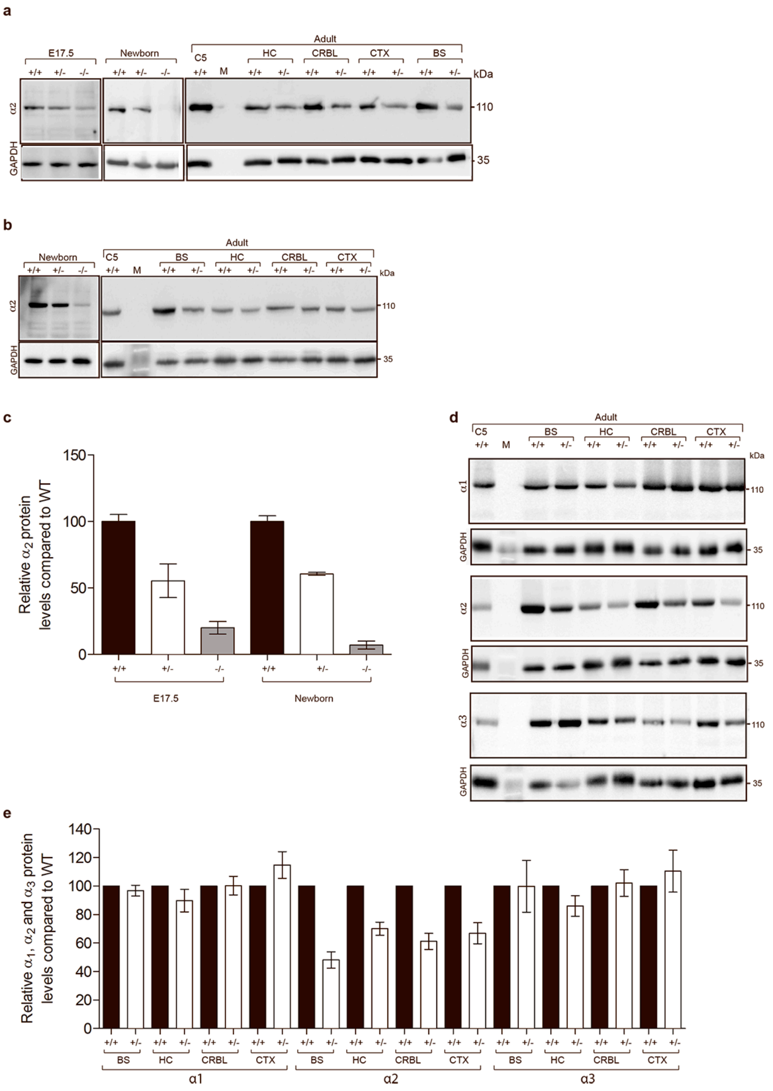

Figure S2. ***In vivo*** **Protein Expression Analysis**

(**a**) Western blot analyses of brain lysates from WT (+/+), heterozygous (+/-) and homozygous (-/-) embryos (E17.5), newborn pups, and various adult male brain lysates, probed with antibodies raised against α_2 (~110 kDa) and glyceraldehyde 3-phosphate dehydrogenase (GAPDH) (~35 kDa) showed reduced α_2 protein level in lysates from embryos/pups/mice harboring a mutation(s). C5; WT adult brain lysate (control), HC; hippocampus, CRBL; cerebellum, CTX; cortex and BS; brain stem. (**b**) Western blot analyses of brain lysates from WT (+/+), heterozygous (+/-) and homozygous (-/-) newborn pups, and various adult female brain lysates, probed with antibodies raised against α_2 (~110 kDa) and glyceraldehyde 3-phosphate dehydrogenase (GAPDH) (~35 kDa) showed reduced α_2 protein level in lysates from pups/mice harboring a mutation(s). (**c**) Densitometric quantification showed reduction in the α_2 protein level in lysates from homozygous and heterozygous E17.5 embryos/newborn pups compared to WT. Error bars represent ± s.e.m (see Supplementary Table 1). (**d**) Western blot analyses of lysates from various adult brain regions, as noted, probed with antibodies raised against α_1, α_2 and α_3 (~110 kDa) and glyceraldehyde 3-phosphate dehydrogenase (GAPDH) (~35 kDa) showed reduced α_2 protein level in lysates, but the α_1 and α_3 protein levels were comparable to WT, for expression ratios see (**e**). For additional screening information and protein quantifications, see Supplementary Table 2.

Abbreviations; C5; WT adult brain lysate (control), HC; hippocampus, CRBL; cerebellum, CTX; cortex, BS; brain stem. M; molecular DNA marker, bp; base pair, H_2O; No template control where DNA was substituted with water.

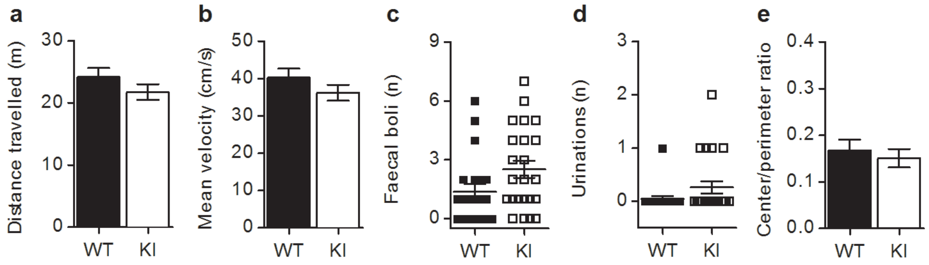

Figure S3. Male $\alpha_2^{+/G301R}$ Mice Behaved as WT in the Open Field Test

(**a,b**) In the open field test, male $\alpha_2^{+/G301R}$ (n=23) and WT (n=21) mice travelled the same distance (m) (**a**) and moved with the same mean velocity (cm/s) (**b**). (**c,d**) Furthermore, male $\alpha_2^{+/G301R}$ and WT mice made the same amount of droppings (n) (**c**) and urinations (n) (**d**). (**e**) In addition, the center/perimeter ratios of male $\alpha_2^{+/G301R}$ and WT mice were comparable.

Data shown as means ± s.e.m.

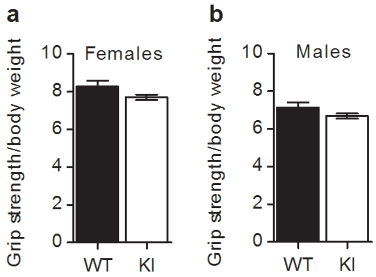

Figure S4. Female and Male $\alpha_2^{+/G301R}$ Mice Had Front Paw Grip Strength Comparable to WT
Body weight (g) and front paw grip strength (g) were determined for each mouse.
(**a,b**) Front paw grip strength/body weight was the same for female (**a**) and male (**b**) $\alpha_2^{+/G301R}$ (n=17 and n=23, respectively) and WT (n=13 and n= 22, respectively) mice.
Data shown as means ± s.e.m.

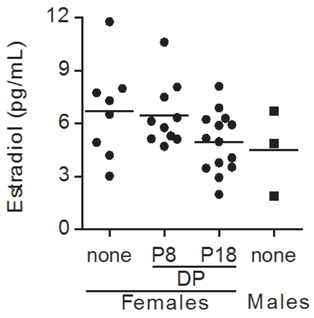

Figure S5. Depoprovera Lowered and Stabilized the Level of Estradiol in Serum from Female WT Mice

Female WT mice (n=24) received Depoprovera (DP)-treatment at the time = 0 days, and after eight (P8) (n=10) or 18 (P18) (n=14) days *post* injection they were decapitated and serum was obtained.

For reference, serum was obtained from untreated female (n=8) and male (n=3) WT mice, and all serum samples were analyzed for the level of estradiol (pg/mL).

Data shown as scatter plots.

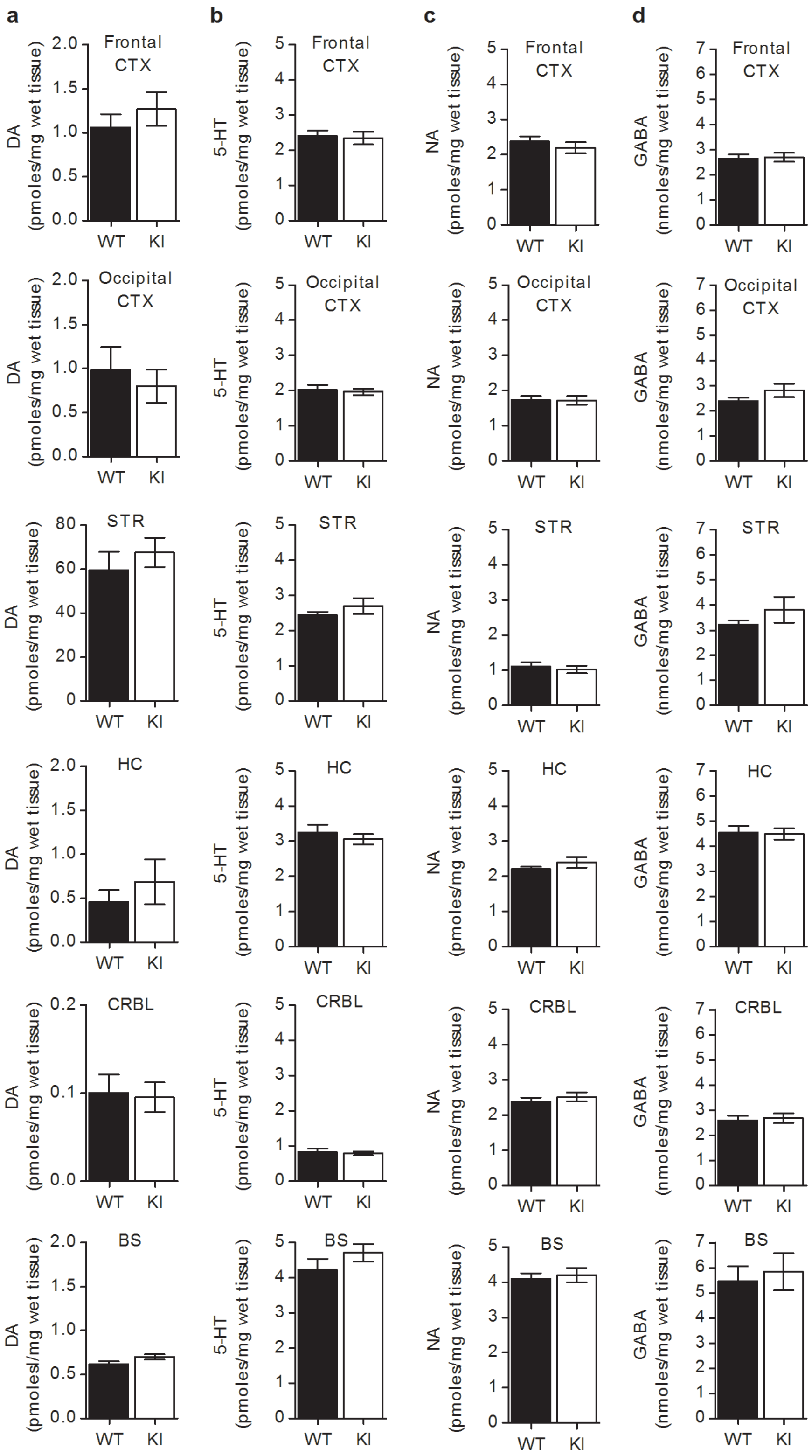

a
b
c
d
Frontal CTX
Occipital CTX
STR
HC
CRBL
BS
DA (pmoles/mg wet tissue)
DA (pmolesl/mg wet tissue)
5-HT (pmoles/mg wet tissue)
NA (pmoles/mg wet tissue)
GABA (nmoles/mg wet tissue)
WT
KI

Figure S6. Comparable Monoamine and GABA Levels Existed in Brain Lysates From $\alpha_2^{+/G301R}$ and WT Mice

(**a-d**) Brain lysates (frontal cortex (CTX), occipital CTX, striatum (STR), hippocampus (HC), cerebellum (CRBL) and brain stem (BS)) were HPLC-analyzed, and $\alpha_2^{+/G301R}$ and WT mice showed comparable levels of dopamine (DA) in CTX (n= 17 and n= 18, respectively), STR (n= 17 and n= 18, respectively), HC (n= 16 and n= 16, respectively), CRBL (n= 5 and n= 6, respectively) and BS (n= 17 and n= 18, respectively) (**a**), serotonin (5-hydroxytryptamine, 5-HT) in CTX (n= 17 and n= 18, respectively), STR (n= 17 and n= 18, respectively), HC (n= 17 and n= 18, respectively), CRBL (n= 16 and n= 17, respectively) and BS (n= 17 and n= 18, respectively) (**b**), noradrenaline (NA) in CTX (n= 17 and n= 18, respectively), STR (n= 17 and n= 18, respectively), HC (n= 17 and n= 18, respectively), CRBL (n= 17 and n= 18, respectively) and BS (n= 17 and n= 18, respectively) (**c**) and gamma-aminobutyric acid (GABA) in CTX (n= 17 and n= 18, respectively), STR (n= 17 and n= 18, respectively), HC (n= 17 and n= 18, respectively), CRBL (n= 17 and n= 18, respectively) and BS (n= 17 and n= 17, respectively) (**d**).

Monoamine levels were expressed as pmoles/mg wet tissue, GABA levels as nmoles/mg wet tissue.

Data shown as means ± s.e.m.

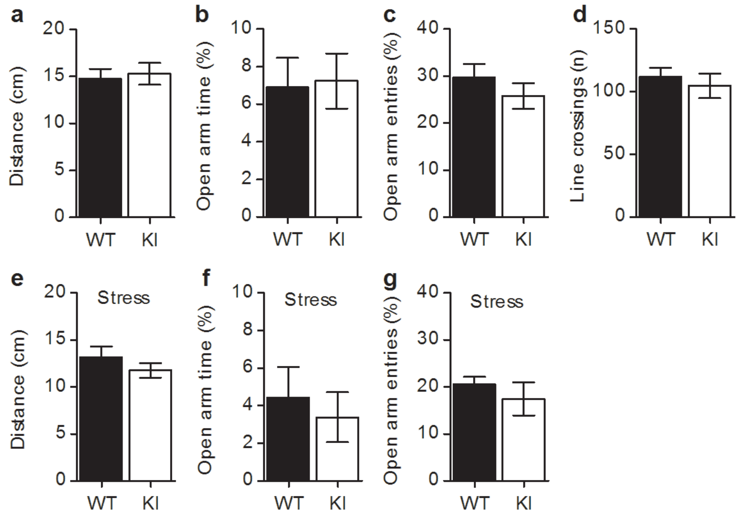

Figure S7. The $\alpha_2^{+/G301R}$ Mice Were Not Anxious in the Elevated Plus Maze Test
(**a-d**) In the elevated plus maze (EPM), $\alpha_2^{+/G301R}$ (n=10) and WT (n=10) mice travelled the same distance (cm) (**a**), spent the same relative amount of time (%) in the open arms (**b**), had the same relative amount of open arm entries (%) (**c**) and had the same number of line crossings (n) (**d**).
(**e-g**) Stress prior to the EPM experiment showed that $\alpha_2^{+/G301R}$ (n=10) and WT (n=10) mice travelled the same distance (cm) (**e**), spent the same relative amount of time (%) in the open arms (**f**) and had the same amount of open arm entries (%) (**g**).
Data shown as means ± s.e.m.
